# Graph Network Feature Space Fusion for Predicting Irregularly Sampled Medical Time-Series Data: Deep Learning Model Development and Validation Study

**DOI:** 10.2196/81145

**Published:** 2026-07-03

**Authors:** Tianle Hong, Zedong Ren, Junfei Fang, Shichao Quan, Jingye Pan, Yezhi Lin

**Affiliations:** 1 Zheyi EENT Hospital of Zhejiang & Eye Ear Nose and Throat Hospital Zhejiang University School of Medicine, Hangzhou, China; 2 The First Affiliated Hospital of Wenzhou Medical University, No. 1, Nanbaixiang Street, Nanbaixiang Sub-district, Ouhai District, Wenzhou, Zhejiang, 325015, China, 86 577 86699816; 3 Department of Big Data in Health Science, The First Affiliated Hospital of Wenzhou Medical University, Wenzhou, China; 4 Zhejiang Engineering Research Center for Hospital Emergency and Process Digitization, Wenzhou, China; 5 Zhejiang Key Laboratory of Critical Care Medicine, Wenzhou, China

**Keywords:** irregularly sampled data, graph network, attention network, clinical phenotype, time-series prediction, multiobjective task

## Abstract

**Background:**

Irregularly sampled data, as a common data structure in the medical field, is frequently observed in emergency clinical datasets. It poses problems such as unequal sampling time intervals and frequencies, making it difficult to align the data without losing information for input into models. Meanwhile, due to information loss in the dataset, it is also difficult for the model to effectively analyze the overall data variation and predict the complex and dynamic conditions of emergency patients in the future.

**Objective:**

This study aimed to address the impact of issues such as missing values and irregularly sampled time intervals on prediction results. This paper proposes a method that converts time-series data into a graph network structure and develops a prediction model for graph data to better support complex tasks.

**Methods:**

In this paper, feature channels and measurement times in time-series data are constructed as nodes, and feature measurements at time points are constructed as edge weights. The point feature space and edge weight space are extracted by a graph convolutional neural network and a gated convolutional attention mechanism network, respectively, and feature fusion is carried out under the action of the learnable head.

**Results:**

The proposed method was tested on 4 datasets and compared with both irregularly sampled time-series predictors and state-of-the-art methods for complete data. In addition, a dedicated analysis was conducted leveraging data from 5 key clinical treatment phases. In single-target classification and single-target regression tasks, the method proposed in this study outperforms other comparative methods in most experiments (accuracy=0.74‐0.88, area under the curve=0.70‐0.87, and mean absolute error=0.038‐0.061). Meanwhile, this study constructs clinical phenotypes during patients’ hospitalization as multitarget prediction tasks, and the proposed model also outperforms comparative methods in multitarget classification tasks (mean accuracy=0.75‐0.91 and mean area under the curve=0.70‐0.75). Ablation experiments verify the effectiveness of fusion between the node feature space and the edge feature space. The interpretability analysis of time points identifies key features that require focused attention for patients across different target tasks and time periods.

**Conclusions:**

The proposed model can effectively analyze medical data with missing values and uneven sampling. It achieves high prediction accuracy in both regression prediction tasks and classification tasks. The graph network-based model structure endows the model with better interpretability, enabling effective exploration of factors that trigger changes.

## Introduction

Irregularly sampled time-series data is a common data pattern in fields such as medicine, meteorology, and transportation. Due to issues such as unequal sampling time intervals and different sampling frequencies between different features, models often use a method of sampling data at certain time intervals to align the overall data queue and fix the time interval of the sequence during the analysis and prediction of irregularly sampled time-series data [1]. For missing values in data series, existing models often use adjacent values or mean values to fill in the gaps [2]. This will cause loss of fine-grained information in time-series data and also discard the time-scale information in time-series data, which will reduce the prediction accuracy of the model when predicting and analyzing complex multiobjective downstream tasks and make it difficult to handle different types of prediction tasks simultaneously. The model constructed in this way is often difficult to solve complex and changing prediction tasks faced in the real world, especially for time-series data analysis in the medical field, which requires models with high prediction accuracy. In the medical field, it is often necessary to perform complex multitask analysis on a large amount of feature data generated in a short period of time for emergency patients, providing information for clinicians to construct treatment plans from multiple dimensions [3]. By constructing targeted treatment plans based on multiple prediction results, the mortality rate of patients can be effectively reduced.

Retrospective studies on patients’ time-series data in medicine have always been an important means to promote the development of basic medicine and explore changes in disease courses. Medical researchers can construct targeted treatment plans by discovering the changing patterns of characteristic data. The analysis methods for time-series data initially used statistical methods to analyze factor changes. With the proposal of machine learning and deep learning algorithms, researchers have built various artificial intelligence models for the classification and regression prediction of medical data [4-6]. However, due to the subjectivity of patients’ willingness to go to the hospital in reality, the sampling time interval of medical time-series data is often uneven, and there are missing values at some time points. At the same time, such data characteristics will also make the model face the problem of uneven input data. In machine learning models, researchers use the method of temporal sampling to align the data. Missing values are processed by zero-value filling or adjacent-value filling. For example, Yoon et al [7] constructed a machine learning model for time-series data of hypotension in ‌intensive care unit patients to explore the relationship between hypotension data and patient mortality. To address the issue of unequal sampling intervals, they aligned the overall data by performing mean sampling every 5 minutes. Delahanty et al [8] predicted the Sequential Organ Failure Assessment scores of patients to assess the onset time of sepsis. For missing data at certain time points, the authors filled in missing values using neighboring values. Wu et al [9] developed a machine learning model to classify the severity levels of chest pain in emergency patients. They used zero padding to handle missing values in the data. While this method introduces a significant amount of noise and homogenizes the original patient features, it allows the model to achieve reasonable accuracy for single-task applications. However, it struggles to maintain high predictive accuracy in multitask scenarios.

With the advancement of model construction methods and the introduction of deep learning algorithm frameworks, the increased flexibility in acceptable input structures has led to the development of various methods for handling missing values in medical time-series data. Lee et al [10] input each patient’s time-series data through three channels: measurement type, measurement value, and measurement behavior. They used an attention mechanism network to fuse and extract overall features, which was applied to predict patient mortality. Bardak and Tan [11] used medical order text data generated from patient clinical treatments to extract features, which were then used to supplement missing values in patients to meet the data requirements for subsequent model training. Xu et al [12] constructed a graph network data structure by applying regular sampling to patient clinical time-series data to address the impact of missing values on model training. They ultimately extracted features and fitted downstream tasks using a gated mechanism and a graph attention network. A comparative analysis of the processing methods of various deep learning models shows different strategies for handling missing values. Encoding the data into a graph network structure improves interpretability and meets the needs of medical research while minimizing the impact of missing values on the model to the greatest extent.

Aiming at the above problems, this paper proposes a data construction method based on a graph network. The feature channels of clinical time-series data of each patient and the recording time points constitute nodes in the graph network. The feature vector of the patient’s feature channel node is composed of the Kullback-Leibler (KL) divergence formed by data distribution in the feature channel, and the generated features are embedded into the feature correlation space for distribution. The patient’s clinical time-series data is input into the model for training after passing the above rule-based diagram network transformation. In the model, the point weight and edge features are extracted from the corresponding space through the model respectively, and fused under the action of a learnable head. The fused features are matched with multiobjective tasks required in clinical practice through the decoder, and the loss function is constructed according to the classification task and the regression task, respectively.

Graph network data structure is a type of data structure that can simultaneously encode spatial information and temporal change information in time-series data, which is widely used in the field of traffic and weather prediction analysis. In the early stage of research, researchers mainly focused on node features and connection relationships for classification and prediction tasks. Lu et al [13] constructed a dual-attention mechanism based on node information in traffic flow data to adaptively predict situations where nodes in the graph network change with time and space simultaneously. Jin et al [14] constructed a set of spatio-temporal prediction model of a graph network for the change of particulate matter 2.5 and predicted the change of node characteristics with time through node clustering in graph network. Sun et al [15] constructed the temperature of the ocean surface as the node feature in the graph network and constructed the transportation direction of ocean currents as the connection edge between nodes and used long short-term memory model to predict the changes of the sea surface temperature.

With the development of more model structures and computer computing power, researchers have added more information such as edge weights, edge connection directions, and point features to the graph network, which improves the final prediction accuracy of the model and can be adapted to more target downstream tasks. For example, Yin et al [16] constructed a dynamic graph network structure for traffic flow data and integrated the edge features and point features in the data into the model at the same time, so that the model could make correct predictions when the network structure changed and better fit the real changes. Feng et al [17] established the graph network of the data at each time point and also established the relationship graph network between different time points, which was used to use the delay data to guide the original data for prediction and analysis. He et al [18] proposed a multilevel fusion spatio-temporal analysis network, which fuses feature extraction from different dimensions in graph networks to adapt to downstream multiobjective tasks. Therefore, this paper uses the data structure of graph network to encode the medical clinical time-series data and makes full use of the point feature space and edge connection weights in the graph network structure to construct the features contained in the clinical time-series data. Based on the data structure of graph network, a time-series analysis and prediction model of multiobjective tasks is constructed to meet the clinical needs.

The data structure of a graph network can retain the fine-grained information and short-term feature change-in information in the patient time-series data to the greatest extent and will not bring additional noise information to the model. The construction of multiobjective tasks can enable the model to meet the needs of clinicians in the process of clinical practice, help doctors predict multidimensional changes in patients’ conditions, and formulate treatment plans for patients. The innovation points proposed in this paper can be summarized as follows:

1. This paper proposes a method to transform medical clinical time-series data into a graph network structure, which preserves the fine-grained information in the data to the greatest extent while reducing the introduction of noise information. The model avoids the problem of missing values and unequal sampling intervals in medical time-series data.
2. This paper proposes a training model for a graph network composed of time-series data, which extracts feature vectors from point feature space and edge weight space, respectively, and fuses them under the action of a learnable head to fit downstream target tasks. Both the classification task and the regression task achieve high prediction accuracy.
3. By extracting feature weights at different time points, this paper provides an interpretable analysis of critical features influencing patient survival over time, thereby assisting clinicians in making targeted and informed decisions at each stage.

## Methods

### Overview

In this section, we will introduce the method of constructing a graph network data structure for time-series data proposed in this paper. In the constructed graph network data, the point features and edge weights are respectively extracted and fused to construct the overall features of the patient time-series graph network. Finally, we will introduce the classifier and the overall model training framework built for the downstream multiobjective task. The overall symbols are summarized in Table 1.

**Table 1.** Explanation of object names used in the calculation formula.

Symbols name	Definition
t	Measurement time
f	Measurement feature
v	Measurement value
$T_{m}$	Calculated time range
$F_{m}$	Calculated number of feature channels
$V_{m}$	Total number of measured behaviors
$P_{t}$	Time node
$P_{f}$	Feature node
$E_{w}$	Connection edge weight
$S_{pf}$	Overall spatial feature set
$F_{b}^{pf}$	Feature vector of a feature node
$S_{pt}$	Overall spatial feature set
$F_{a}^{pt}$	Feature vector of a time feature node
$X_{f}$	Feature matrix of the nodes
$A_{w}$	Edge weight matrix
$F_{p}$	Overall feature of the node feature space
$W_{E}$	Feature value matrix
$W_{EF}$	Partially forgotten feature matrix
$A_{E}$	Output of multihead attention mechanism layer
$F_{e}$	Feature obtained from the edge weight space
$F_{total}$	Final total features

### Graph Network Construction

To retain the fine-grained information in the patient time-series data to the greatest extent and reduce the introduction of noise information, this paper constructs the clinical time-series data as a graph network structure to input into the model calculation. In the construction of the graph network data structure, the feature space of nodes is first constructed. By disassembling the clinical time-series data, it is found that each data measurement behavior can be divided into three dimensions: measurement time (*t*), measurement feature (*f*), and measurement value (*v*). Based on the calculated time range (*T*_m_), the calculated number of feature channels (*F*_m_), the total number of measured behaviors (V_m_), and the overall time-series data of the patient can be expressed as $\{(t_{1},f_{1},v_{1}),(t_{1},f_{2},v_{2}),....(t_{a},f_{b},v_{c})\}a\leq T_{m},b\leq F_{m},c\leq V_{m}$. Among them, the measurement time and the measurement feature constitute the time node (P_t_) and the feature node (P_f_) in the graph network data, respectively, and the measurement value is constructed as the connection edge weight (E_w_) between the time node and the feature node. The feature space of the feature node (P_f_) is mapped in the feature correlation space and composed of the KL divergence values calculated between the original data within the computational time range across the F_m_ feature channels of each patient. This study references multiple studies on feature space construction based on KL divergence [19,20]. The use of KL divergence to measure distributional differences between distinct physiological variables (eg, heart rate and temperature) may indeed appear counterintuitive from a clinical perspective. However, it is important to clarify that KL divergence is not used here to infer causal or biological relationships between different features. Instead, it serves as a similarity metric to construct the feature node embeddings in the graph network. Specifically, it quantifies the distributional alignment between different physiological time series within the same patient, which may reflect the degree of systemic physiological integration or dysregulation—a key characteristic of critical illness progression. This approach is supported by prior work in multivariate time-series analysis, where distributional similarity is used to capture latent coordination among physiological systems. The specific construction method is as follows: first, select feature *f*; second, compute the KL divergence values between feature (f) and all other features to form the point feature vector for this feature; repeat this process until the point feature vectors for all features are constructed. The feature distributions used for a specific prediction time point are calculated strictly using data recorded strictly before that (T_m_) time. For example, when constructing the graph network for prediction at time (T_m_), only measurements with time stamps < T_m_ are used to compute the KL divergence and edge weights. No future information is incorporated, thereby preventing temporal data leakage. The feature space integrates the information correlation and distribution correlation between feature channels and establishes the relationship between the edge weight value and the point feature space. At the same time, the KL divergence values in the overall feature space are normalized to ensure that the feature distribution is in the feature correlation space and reduce the impact on model training. The calculation method of the feature space (P_f_) is as follows:


(1) $$S_{pf}=\{F_{1}^{pf},F_{2}^{pf}......F_{b}^{pf}\}b\leq F_{m}$$

(2) $$F_{b}^{pf}=\left[KL\left(f_{b},f_{1}\right),KL\left(f_{b},f_{2}\right).......KL\left(f_{b},f_{F_{m}}\right)\right]\;b\leq F_{m}$$

Probability distribution construction for KL divergence under sparse data: for each feature channel, let its available measurements before prediction time be $\left\{v_{1},v_{2},...v_{n}\right\}$. This paper constructs a discrete probability distribution over a fixed set of M bins spanning the global range of all features. When n=2, we use kernel density estimation with a Gaussian kernel; when n=1, the distribution is a Gaussian centered at that single value; and when n=0, we assign a uniform distribution. The KL divergence between 2 features is then computed on these discrete distributions. Where, S_pf_ represents the overall spatial feature set, and F^pf^_b_ represents the feature vector of a feature node. Meanwhile, the feature space mapping of the time mode P_t_ is in the temporal difference embedding space, built by the difference between the time node and the measured time of the remaining nodes. The feature space is used to reflect the correlation of the time values in the measured time series. The calculation time point is strictly limited within the calculation time range. The information provided by the time node can assist the model in mining the time scale between different measurements and provide the change information of the time scale for the model training. The calculation method of the feature space P_t_ is as follows:


 (3) $$S_{pt}=\left\{F_{1}^{pt},F_{2}^{pt},\ldots ,F_{a}^{pt}\right\},\;a\leq T_{m}$$

(4) $$\begin{array}{r} F_{a}^{pt}=[-\left\|\left(t_{a},t_{1}\right)\right\|,-\left\|\left(t_{a},t_{2}\right)\right\|,\ldots ,\\ -\left\|\left(t_{a},t_{m}\right)\right\|],\;a\leq T_{m} \end{array}$$

**Figure 1.**
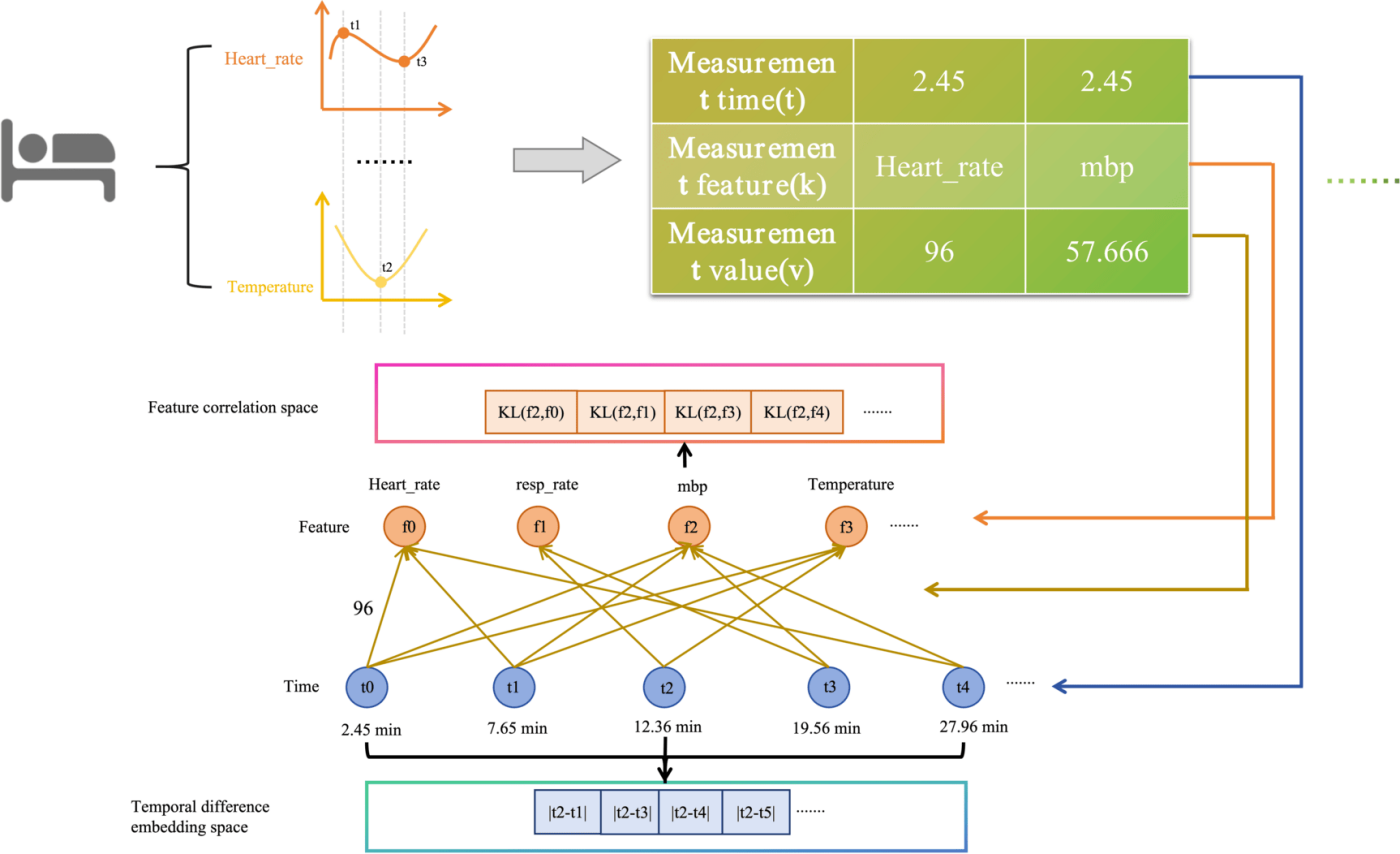
Graph network data structure construction figure: the feature channels and time in each patient’s clinical time-series data record constitute nodes, and the record values constitute edge weights. The Kullback-Leibler (KL) divergence and the time difference form the point features.

Where, S_pt_ represents the overall spatial feature set, and F^pt^_a_ represents the feature vector of a time feature node. The connection edge E_w_ of the graph network is composed of v_c_ in the measurement behavior, and the existence of the connection edge is directly related to the existence of the measurement behavior. In this way, the fine-grained information in the data is retained to the greatest extent, and the introduction of noise information is eliminated. The construction method of the graph network is shown in Figure 1.

### Model Construction

After constructing a graph network with time-series data, this paper proposes the construction of a graph convolutional network (GCN) by using the point feature space and the edge weight connection relationship to extract the overall features in the point feature space. At the same time, a multihead attention mechanism network with a gated mechanism connecting multiple layers is proposed for feature extraction in the edge weight feature space. Finally, a learnable weight head is added to the features of the 2 dimensions for feature fusion to meet the downstream multiobjective tasks.

First, in the GCN, the feature spaces S_pf_ and S_pt_ are jointly constructed as the feature matrix X_f_ of the nodes. The collected values of each feature for an individual patient at different time points are normalized, and the connection values corresponding to time nodes and feature nodes form the edge weight matrix A_w_, and the learnable parameter Wis added. The GCN [21] network with L) layer input edge weights is used to construct and calculate the overall feature F_p_ of the node feature space. The calculation is as follows:


(5) $$H(1)=\sigma (A_{w}X_{f}W_{0})$$

(6) $$F_{p}=H\left(L\right)=\sigma \left(A_{w}H\left(L-1\right)\;W_{L-1}\right)$$

Second, during the computation process of the GCN network, the model primarily focuses on the intrapatient variations, and the edge features tend to gradually weaken as the training depth increases. To fully express the features in the edge weight space, this paper proposes a targeted approach. First, each feature of the edge weight (E_w_) is independently normalized across the entire set of patient samples. This normalized edge weight data is then used to construct the feature value matrix (W_E_). Finally, (W_E_) is input into the convolutional gated attention mechanism network.

During the computation process of the convolutional gated attention mechanism network, first, W_E_ is fed into the forgetting gate to construct the partially forgotten feature matrix (W_EF_). Then, W_EF_ and W_E_ are input into the 2D convolution layer at the same time, and the multidimensional data (C_EF_) and (C_E_) are generated after convolution and input into the multihead attention mechanism layer [22] to obtain A_E_(1). It is constructed as shown in the following formula:


(7) $$W_{EF}=Sigmoid\left(h_{1}W_{E}+b_{1}\right)$$

(8) $$C_{EF}=Conv2d\left(W_{EF}\right)$$

(9) $$C_{E}=Conv2d(W_{E})$$

(10) $$A_{E}(1)=Attention(C_{EF},C_{E},C_{E})$$

Here, (h_1_) represents the weight matrix of the first layer and (b_1_) represents the bias term of the first layer. The feature A_E_(1) obtained from the first layer is reinput into the convolutional gated network to generate the C_AE_ after passing through the average pooling layer. C_AE_, A_E_(1), and C_E_ are again input into the multihead attention mechanism layer. Repeat the operation of feature extraction through (L) layers, and finally obtain the extracted feature weight (F_e_) of the edge feature space. The overall calculation formula is as follows:


(11) $$\begin{array}{r} C_{AE}(L)=Conv2d(Sigmoid(h_{L}\;AveragePooling(A_{E}(L-1))\\ +b_{L})) \end{array}$$

(12) $$A_{E}\left(L\right)=Attention\left(C_{AE}\left(L\right),A_{E}\left(L-1\right),C_{E}\right)$$

(13) $$F_{e}=AveragePooling\left(A_{E}\left(L\right)\right)$$

Finally, the feature (F_p_) obtained from the point feature space and the feature (F_e_) obtained from the edge weight space were added to the learnable weight head (H_fp_) and (H_fe_), respectively, to form the overall feature extracted from the time-series data graph network space. The features are computed as follows:


(14) $$F_{total}=H_{fp}F_{p}+H_{fe}F_{e}$$

### Model Training

After obtaining the overall features extracted from the temporal graph network, we build different decoders to satisfy the downstream multiobjective task. For the classification prediction task, it is necessary to trace the overall feature changes of patients and conduct a comprehensive analysis. For the 2 different classification tasks of patient mortality and patient clinical representation, the overall feature matrix (F_total_) is decomposed and arranged by time and mapped to the corresponding dimension through the feedforward neural network for training. The binary cross-entropy loss function is used to calculate the task loss value, and the calculation formula is as follows:


(15) $$\begin{array}{rl} BceLoss & =-\frac{1}{N}\sum_{i=0}^{N}y_{i}\;\log(p(y_{i}))\\ \;+(1-y_{i})\;\log(1-y_{i}) \end{array}$$

For the regression task, the key consideration is the feature vector of patients at the final time point. For regression prediction tasks such as predicting the future length of hospital stay or the future change of different physical sign data, this paper uses the features of the last dimension of the point feature space and edge weight space, which are weighted by H_fp_ and H_fe_ and then mapped to the corresponding dimension by the feedforward neural network for training. The calculation formula is as follows:


(16) $$L1loss=\frac{1}{N}\sum_{i=0}^{N}\left|y_{i}-f(y_{i})\right|$$

The length of hospital stay was normalized before model training using minimum-maximum scaling. Specifically, let (y_st_) denote the original stay time in hours. The normalized value (y_norm_) is computed as:


$$y_{norm}=\frac{y_{st}-y_{min}}{y_{max}-y_{min}}$$

**Figure 2.**
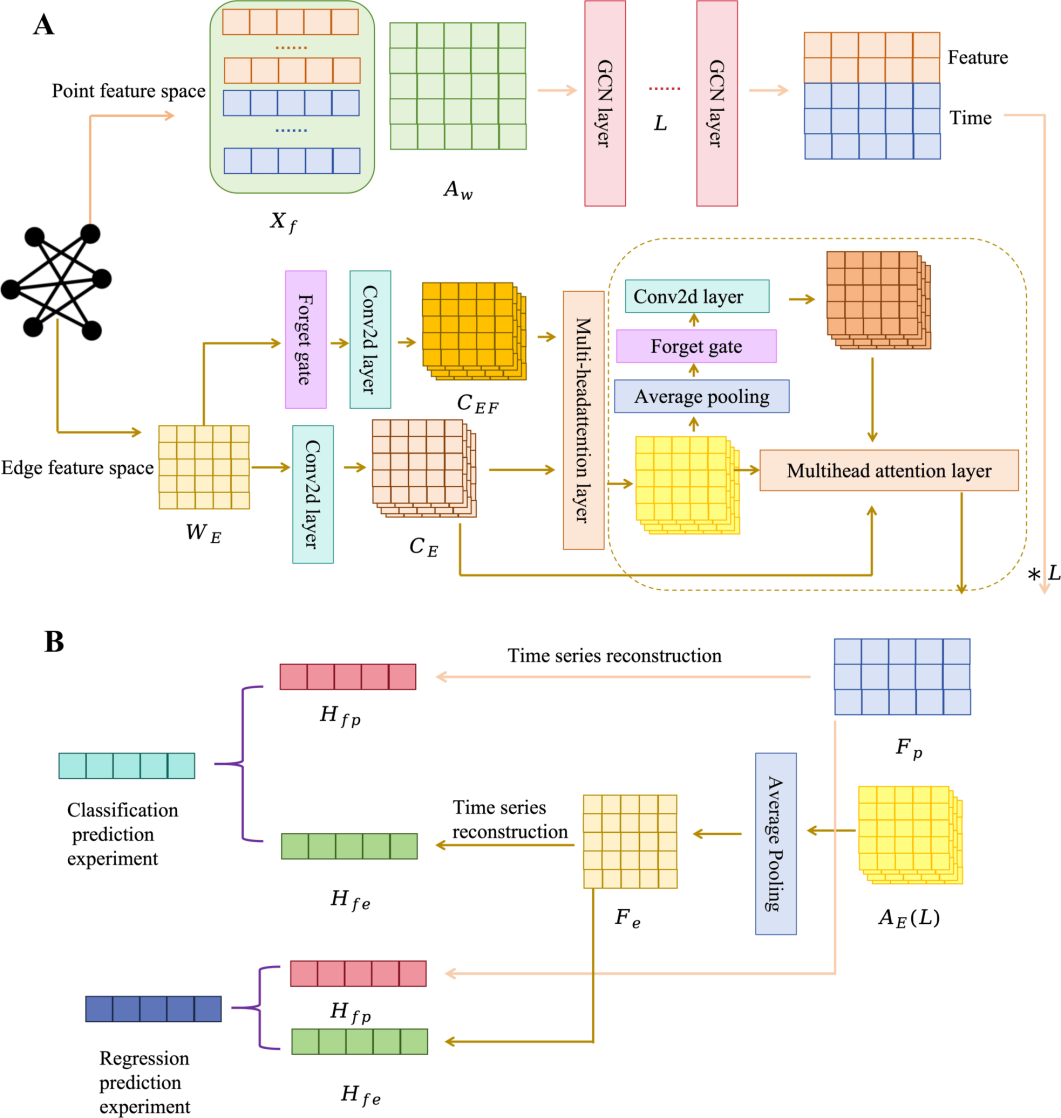
Model and training structure figure. (A) Features are extracted by building models for the point feature space and the edge weight space, respectively. A multilayer graph convolutional network (GCN) is used for the point feature space, and a multilayer gated convolutional attention mechanism layer is used for the edge weight space. (B) Feature fusion and extraction of different dimensions of data for training downstream tasks. Here, * represents the multiplication sign, X_f_ represents the feature matrix of the nodes, A_w_ represents the edge weight matrix, L represents the number of GCN layers, W_E_ represents the feature value matrix. C_E_ represents the matrix obtained by convolving the variable weight matrix, C_EF_ represents the matrix obtained by convolving and applying the forget gate to the variable weight matrix, H_fp_ and H_fe_ represent temporal features extracted from graph network data, F_p_ represents feature matrix constructed based on graph network data, F_e_ represents feature matrix constructed based on edge weight data, and A_E_(L) represents feature matrix obtained after the L-th GCN layer.

The overall model construction and training are shown in Figures 2A and 2B.

### Ethical Considerations

This research was conducted in accordance with the Declaration of Helsinki and was approved by the Ethics Committee of the First Affiliated Hospital of Wenzhou Medical University (approval KY2023-R061). The requirement for written informed consent was waived by the Institutional Review Board due to the retrospective nature of the study. The study data are anonymous or deidentified. Meanwhile, no identification of individual images in the paper or additional material is possible.

## Results

### Dataset Introduction

In the experimental data part of this paper, the time-series data of patients extracted from the public datasets Medical Information Mart for Intensive Care (MIMIC) III [23,24] and MIMIC-IV [25] are used. Under the guidance of the clinical practice experience of clinicians, this paper selects 42 important features of concern in clinical practice for model construction. Meanwhile, the patient’s survival, length of hospital stay, and clinical phenotypes are constructed as the outcome variables of the model. Since the recording time of the MIMIC-IV dataset is in the transition period of characterizing the coding rules, it is split into two parts: *ICD-9 (International Classification of Diseases, 9th Revision)* and *ICD-10* (*International Classification of Diseases, 10th Revision)*. The data of patients collected by the Critical Care Medicine Department of the First Affiliated Hospital of Wenzhou Medical University from 2012 to 2021 are also used to verify the effectiveness of the model. The data of 2000 patients in each dataset are selected in a 1:1 ratio of death to survival to explore the effectiveness of the proposed model in a balanced and fair way. Overall statistics for the data are provided in Table 2. Among them, the format of categorical data is expressed as “number of patients to number of nonpatients,” and the format of numerical data is expressed as “mean (SD).” To verify the stability and effectiveness of the model, the proposed model was evaluated on an expanded dataset of 4000 samples with a 1:1 ratio of death to survival, analyzing temporal feature data more than 6-hour and 12-hour time windows. The results were compared with those of state-of-the-art methods, and the detailed comparison is provided in Multimedia Appendix 1.

**Table 2.** Statistical summary of patient outcome measures.

Classification of diseases	*ICD-9^a^*		*ICD-10* ^b^	
Label name	MIMIC-III^c^	MIMIC-IV^d^	MIMIC-IV	Private data
Death (positive cases/negative cases)	1000/1000	1000/1000	1000/1000	1000/1000
Congestive heart failure (positive cases/negative cases)	774/1226	1076/924	886/1114	971/1039
Essential hypertension (positive cases/negative cases)	785/1215	824/1176	736/1264	557/1443
Atrial fibrillation (positive cases/negative cases)	721/1279	734/1266	883/1117	552/1448
ARDS^e^ (positive cases/negative cases)	574/1426	614/1386	630/1370	368/1632
Coronary atherosclerosis (positive cases/negative cases)	558/1442	589/1411	522/1478	348/1652
Acute kidney failure (positive cases/negative cases)	495/1505	534/1466	755/1245	278/1722
Urinary tract infection (positive cases/negative cases)	413/1587	478/1512	402/1598	274/1726
Diabetes mellitus (positive cases/negative cases)	378/1622	498/1502	423/1577	279/1721
Anaphylactic shock (positive cases/negative cases)	348/1652	654/1346	274/1726	176/1824
Pneumonia (positive cases/negative cases)	325/1675	477/1523	493/1507	151/1849
Sepsis (positive cases/negative cases)	322/1678	343/1657	654/1346	127/1873
Pneumonitis (positive cases/negative cases)	328/1672	350/1650	739/1261	148/1852
Atelectasis (positive cases/negative cases)	302/1698	286/1714	338/1662	162/1838
Myocardial infarction (STEMI^f^; positive cases/negative cases)	256/1744	269/1731	675/1325	129/1871
Familial hypercholesterolemia (positive cases/negative cases)	235/1765	387/1613	322/1678	110/1890
COPD^g^ (positive cases/negative cases)	219/1781	214/1786	436/1564	131/1869
Liver failure (positive cases/negative cases)	245/1755	772/1228	792/1208	150/1850
Metabolic acidosis (positive cases/negative cases)	232/1768	244/1756	269/1731	121/1879
Anemia (positive cases/negative cases)	234/1766	299/1701	226/1774	113/1887
Septic shock (positive cases/negative cases)	226/1774	304/1696	323/1674	124/1876
Hypertensive renal disease (positive cases/negative cases)	172/1828	173/1827	272/1728	123/1877
Pleural effusion (positive cases/negative cases)	202/1798	214/1786	567/1433	124/1876
Iron deficiency anemia (positive cases/negative cases)	189/1811	200/1800	221/1779	121/1879
Cardiogenic shock (positive cases/negative cases)	183/1817	258/1742	394/1606	120/1880
Hypothyroidism (positive cases/negative cases)	154/1846	248/1752	281/1719	113/1887
Complications of surgical procedures on the circulatory system (positive cases/negative cases)	182/1818	273/1727	355/1645	94/1906
Aortic insufficiency (positive cases/negative cases)	166/1834	478/1522	217/1783	105/1895
ITP^h^ (positive cases/negative cases)	163/1837	202/1798	254/1746	104/1896
GERD^i^ (positive cases/negative cases)	143/1857	220/1780	380/1620	65/1935
Cardiac arrest (positive cases/negative cases)	149/1851	389/1611	256/1744	81/1919
Stay time (hours), mean (SD)	262.4 (272.7)	279.8 (283.0)	311.6 (339.3)	508.7 (490.1)

a *ICD-9*: *International Classification of Diseases, 9th Revision.*

b *ICD-10*: *International Classification of Diseases, 10th Revision.*

c MIMIC-III: Medical Information Mart for Intensive Care III.

d MIMIC-IV: Medical Information Mart for Intensive Care IV.

e ARDS:‌ acute respiratory distress syndrome.

f STEMI: ST-segment elevation myocardial infarction.

g COPD: chronic obstructive pulmonary disease.

h ITP: immune thrombocytopenia.

i GERD:‌ gastroesophageal reflux disease.

### Classification Prediction Experiment

In this paper, the proposed model is compared with advanced time-series prediction models in two single- and multi-objective tasks: predicting the final death of a patient and predicting whether multiple phenotypes of a patient occur. The study explores the advantages and scope of application of the proposed model. The experimental equipment adopted in this paper includes an Intel(R) Xeon(R) Silver 4210R CPU running at 2.40 GHz, an NVIDIA GeForce RTX 3090 graphics card with 24 GB of video memory, and 16 GB of system memory. The proposed model in this paper is compared with state-of-the-art time-series prediction models designed for irregularly sampled data, including time-aware long short-term memory [26], gated recurrent unit with decay [27], multitime attention networks for irregularly sampled time series [28], and ContiFormer [29]. Additionally, we adopt time-series analysis methods such as fully convolutional networks [30], temporal convolutional networks [31], neural basis expansion analysis for interpretable time series forecasting [32], Crossformer [33], and DSformer [34], which are trained on regularly sampled data with missing values imputed by the mean. The final comparison results provide a more multidimensional perspective for evaluating time-series prediction performance. For the time range of data length, this paper refers to the experience in the clinical practice process to construct 5 important time period lengths for training and comparison. The hyperparameter tuning of the model refers to multiple state-of-the-art hyperparameter optimization methods [35-39], and the optimal training parameters for each model are obtained. Detailed data are provided in Multimedia Appendix 2. The accuracy and area under the curve for predicting death for each model are provided in Tables3  4.

**Table 3.** Accuracy results for mortality prediction using the proposed method and advanced time-series methods across 4 datasets.

Data and method	6 hours	12 hours	24 hours	36 hours	48 hours
MIMIC^a^-III					
FCN^b^	0.55 (0.01)	0.51 (0.00)	0.54 (0.02)	0.68 (0.01)	0.73 (0.01)
TCN^c^	0.68 (0.01)	0.73 (0.00)	0.70 (0.04)	0.74 (0.01)	0.78 (0.02)
N-BEATS^d^	0.56 (0.05)	0.60 (0.05)	0.62 (0.01)	0.60 (0.00)	0.68 (0.00)
Crossformer	0.71 (0.05)	0.74 (0.03)	0.70 (0.03)	0.73 (0.03)	0.78 (0.02)
DSformer	0.72 (0.02)	0.74 (0.02)	0.71 (0.03)	0.68 (0.02)	0.60 (0.06)
T-LSTM^e^	0.57 (0.01)	0.56 (0.01)	0.56 (0.01)	0.59 (0.01)	0.57 (0.01)
GRU-D^f^	0.56 (0.01)	0.57 (0.00)	0.56 (0.01)	0.60 (0.01)	0.58 (0.02)
mTAND^g^	0.56 (0.01)	0.58 (0.00)	0.57 (0.01)	0.55 (0.01)	0.57 (0.01)
ContiFormer	0.75 (0.03)	0.72 (0.01)	0.74 (0.01)	0.72 (0.04)	0.74 (0.04)
Our work	0.76 (0.03)	0.76 (0.02)	0.74 (0.00)	0.78 (0.01)	0.81 (0.01)
MIMIC-IV^h^ *ICD-9^i^*					
FCN	0.84 (0.02)	0.72 (0.01)	0.77 (0.02)	0.80 (0.02)	0.76 (0.00)
TCN	0.77 (0.01)	0.79 (0.02)	0.84 (0.01)	0.86 (0.02)	0.80 (0.00)
N-BEATS	0.76 (0.03)	0.70 (0.01)	0.75 (0.02)	0.83 (0.02)	0.76 (0.03)
Crossformer	0.80 (0.08)	0.80 (0.04)	0.84 (0.04)	0.80 (0.04)	0.82 (0.02)
DSformer	0.81 (0.03)	0.80 (0.02)	0.77 (0.02)	0.83 (0.01)	0.82 (0.03)
T-LSTM	0.73 (0.02)	0.73 (0.01)	0.74 (0.01)	0.73 (0.01)	0.76 (0.02)
GRU-D	0.73 (0.00)	0.75 (0.01)	0.76 (0.02)	0.76 (0.00)	0.78 (0.00)
mTAND	0.75 (0.00)	0.74 (0.01)	0.76 (0.01)	0.81 (0.00)	0.78 (0.02)
ContiFormer	0.73 (0.00)	0.72 (0.00)	0.68 (0.02)	0.67 (0.05)	0.72 (0.00)
Our work	0.86 (0.03)	0.86 (0.01)	0.88 (0.02)	0.86 (0.01)	0.84 (0.04)
MIMIC-IV *ICD-10^j^*					
* FCN*	0.77 (0.00)	0.73 (0.00)	0.82 (0.02)	0.80 (0.01)	0.80 (0.02)
TCN	0.82 (0.02)	0.86 (0.02)	0.80 (0.01)	0.85 (0.02)	0.82 (0.00)
N-BEATS	0.84 (0.01)	0.77 (0.01)	0.85 (0.03)	0.83 (0.01)	0.83 (0.02)
Crossformer	0.85 (0.03)	0.84 (0.04)	0.80 (0.02)	0.83 (0.06)	0.85 (0.01)
DSformer	0.84 (0.02)	0.85 (0.03)	0.81 (0.00)	0.85 (0.06)	0.80 (0.07)
T-LSTM	0.65 (0.01)	0.66 (0.02)	0.67 (0.01)	0.66 (0.01)	0.71 (0.02)
GRU-D	0.67 (0.00)	0.68 (0.02)	0.68 (0.01)	0.72 (0.01)	0.73 (0.03)
mTAND	0.69 (0.01)	0.70 (0.01)	0.70 (0.01)	0.73 (0.01)	0.74 (0.01)
ContiFormer	0.64 (0.03)	0.69 (0.01)	0.69 (0.02)	0.66 (0.06)	0.75 (0.01)
Our work	0.85 (0.02)	0.86 (0.03)	0.87 (0.01)	0.88 (0.04)	0.85 (0.02)
Private dataset					
FCN	0.65 (0.01)	0.68 (0.01)	0.53 (0.01)	0.60 (0.02)	0.55 (0.01)
TCN	0.65 (0.01)	0.68 (0.01)	0.66 (0.00)	0.63 (0.00)	0.63 (0.00)
N-BEATS	0.67 (0.01)	0.64 (0.02)	0.60 (0.05)	0.64 (0.04)	0.70 (0.01)
Crossformer	0.64 (0.03)	0.65 (0.02)	0.63 (0.03)	0.67 (0.05)	0.70 (0.01)
DSformer	0.69 (0.02)	0.69 (0.03)	0.72 (0.02)	0.70 (0.01)	0.68 (0.02)
T-LSTM	0.59 (0.01)	0.62 (0.01)	0.63 (0.02)	0.62 (0.01)	0.64 (0.01)
GRU-D	0.60 (0.00)	0.61 (0.00)	0.64 (0.01)	0.62 (0.01)	0.64 (0.01)
mTAND	0.56 (0.01)	0.60 (0.00)	0.62 (0.03)	0.62 (0.01)	0.63 (0.01)
ContiFormer	0.70 (0.06)	0.74 (0.01)	0.75 (0.00)	0.79 (0.07)	0.68 (0.06)
Our work	0.69 (0.01)	0.75 (0.02)	0.77 (0.01)	0.73 (0.02)	0.74 (0.01)

a MIMIC-III: Medical Information Mart for Intensive Care III.

b FCN: fully convolutional network.

c TCN: temporal convolutional network.

d N-BEATS: neural basis expansion analysis for interpretable time series forecasting.

e T-LSTM: time-aware long short-term memory.

f GRU-D: gated recurrent unit with decay.

g mTAND: multitime attention networks for irregularly sampled time series.

h MIMIC-IV: Medical Information Mart for Intensive Care IV.

i *ICD-9*: *International Classification of Diseases, 9th Revision.*

j ICD-10: *International Classification of Diseases, 10th Revision.*

**Table 4.** Area under the curve results for mortality prediction using the proposed method and advanced time-series methods across 4 datasets.

Data and method	6 hours	12 hours	24 hours	36 hours	48 hours
MIMIC-III^a^					
FCN^b^	0.53 (0.01)	0.52 (0.01)	0.55 (0.01)	0.60 (0.02)	0.68 (0.02)
TCN^c^	0.66 (0.02)	0.68 (0.00)	0.68 (0.01)	0.69 (0.01)	0.66 (0.01)
N-BEATS^d^	0.57 (0.02)	0.62 (0.00)	0.64 (0.01)	0.63 (0.02)	0.63 (0.01)
Crossformer	0.68 (0.02)	0.67 (0.02)	0.66 (0.01)	0.67 (0.02)	0.67 (0.02)
DSformer	0.69 (0.00)	0.68 (0.03)	0.68 (0.02)	0.65 (0.03)	0.61 (0.03)
T-LSTM^e^	0.58 (0.00)	0.57 (0.02)	0.57 (0.01)	0.61 (0.01)	0.57 (0.00)
GRU-D^f^	0.60 (0.02)	0.58 (0.00)	0.58 (0.02)	0.61 (0.02)	0.61 (0.03)
mTAND^g^	0.56 (0.01)	0.58 (0.01)	0.56 (0.01)	0.59 (0.01)	0.59 (0.01)
ContiFormer	0.60 (0.00)	0.66 (0.00)	0.66 (0.01)	0.68 (0.01)	0.67 (0.01)
Our work	0.70 (0.02)	0.74 (0.00)	0.73 (0.03)	0.73 (0.01)	0.78 (0.02)
MIMIC-IV^h^ *ICD-9^i^*					
FCN	0.79 (0.01)	0.75 (0.03)	0.76 (0.01)	0.78 (0.00)	0.78 (0.01)
TCN	0.78 (0.02)	0.80 (0.00)	0.85 (0.01)	0.85 (0.03)	0.80 (0.02)
N-BEATS	0.77 (0.01)	0.72 (0.02)	0.74 (0.03)	0.76 (0.02)	0.77 (0.01)
Crossformer	0.79 (0.02)	0.81 (0.01)	0.80 (0.00)	0.81 (0.02)	0.80 (0.01)
DSformer	0.80 (0.01)	0.78 (0.01)	0.78 (0.00)	0.79 (0.02)	0.83 (0.02)
T-LSTM	0.80 (0.00)	0.79 (0.00)	0.79 (0.01)	0.79 (0.01)	0.83 (0.00)
GRU-D	0.78 (0.01)	0.80 (0.01)	0.81 (0.02)	0.82 (0.00)	0.85 (0.00)
mTAND	0.81 (0.01)	0.78 (0.00)	0.80 (0.01)	0.81 (0.00)	0.83 (0.01)
ContiFormer	0.65 (0.00)	0.66 (0.02)	0.66 (0.00)	0.67 (0.01)	0.67 (0.00)
Our work	0.84 (0.02)	0.85 (0.02)	0.86 (0.01)	0.87 (0.01)	0.86 (0.03)
MIMIC-IV *ICD-10^j^*					
FCN	0.70 (0.01)	0.71 (0.00)	0.72 (0.01)	0.70 (0.01)	0.71 (0.02)
TCN	0.76 (0.02)	0.75 (0.01)	0.76 (0.00)	0.77 (0.00)	0.76 (0.01)
N-BEATS	0.75 (0.01)	0.74 (0.02)	0.75 (0.01)	0.73 (0.02)	0.78 (0.00)
Crossformer	0.78 (0.03)	0.79 (0.02)	0.76 (0.02)	0.77 (0.03)	0.79 (0.02)
DSformer	0.76 (0.01)	0.75 (0.01)	0.75 (0.00)	0.76 (0.02)	0.74 (0.01)
T-LSTM	0.68 (0.01)	0.71 (0.00)	0.70 (0.00)	0.71 (0.00)	0.78 (0.00)
GRU-D	0.72 (0.00)	0.73 (0.02)	0.70 (0.01)	0.77 (0.00)	0.79 (0.00)
mTAND	0.73 (0.01)	0.74 (0.01)	0.73 (0.01)	0.77 (0.01)	0.79 (0.00)
ContiFormer	0.63 (0.02)	0.65 (0.00)	0.65 (0.01)	0.66 (0.00)	0.66 (0.00)
Our work	0.78 (0.02)	0.80 (0.03)	0.82 (0.01)	0.81 (0.03)	0.83 (0.02)
Private dataset					
FCN	0.66 (0.02)	0.66 (0.00)	0.60 (0.01)	0.62 (0.02)	0.59 (0.03)
TCN	0.64 (0.02)	0.63 (0.01)	0.65 (0.01)	0.64 (0.01)	0.65 (0.01)
N-BEATS	0.67 (0.01)	0.65 (0.02)	0.64 (0.04)	0.66 (0.03)	0.68 (0.01)
Crossformer	0.67 (0.01)	0.68 (0.02)	0.65 (0.05)	0.69 (0.03)	0.70 (0.01)
DSformer	0.67 (0.00)	0.66 (0.01)	0.69 (0.04)	0.70 (0.02)	0.70 (0.03)
T-LSTM	0.62 (0.01)	0.64 (0.01)	0.67 (0.01)	0.64 (0.02)	0.68 (0.01)
GRU-D	0.61 (0.01)	0.63 (0.00)	0.69 (0.01)	0.65 (0.01)	0.68 (0.00)
mTAND	0.62 (0.02)	0.63 (0.01)	0.67 (0.01)	0.67 (0.01)	0.68 (0.01)
ContiFormer	0.68 (0.01)	0.68 (0.01)	0.71 (0.01)	0.64 (0.05)	0.70 (0.01)
Our work	0.70 (0.01)	0.73 (0.01)	0.74 (0.02)	0.70 (0.01)	0.75 (0.02)

a MIMIC-III: Medical Information Mart for Intensive Care III.

b FCN: fully convolutional network.

c TCN: temporal convolutional network.

d N-BEATS: neural basis expansion analysis for interpretable time series forecasting.

e T-LSTM: time-aware long short-term memory.

f GRU-D: gated recurrent unit with decay.

g mTAND: multitime attention networks for irregularly sampled time series.

h MIMIC-IV: Medical Information Mart for Intensive Care IV.

i *ICD-9*: *International Classification of Diseases, 9th Revision.*

j *ICD-10*: *International Classification of Diseases, 10th Revision.*

From the results, it can be seen that the proposed model is in a leading position in most tasks of the 4 datasets, and the results of the proposed method are equal to or slightly inferior to the results of the comparison methods in some time periods of some datasets. Meanwhile, the proposed model demonstrates accuracy in predicting mortality on a larger-scale dataset. The detailed comparison results are provided in Multimedia Appendix 1. From the analysis, it can be found that death is a simple binary classification task, and most of the time-series analysis methods can effectively capture the change law of time-series data to make the correct judgment. The wrong part of the judgment may be due to changes in the patient’s condition, resulting in the patient’s condition getting better or suddenly worse. Moreover, in the public datasets, the clinical time-series data of patients are collected with high frequency and high data density, and the results of various time-series prediction methods are basically similar. However, in the private dataset, due to different clinical policies, the data collection frequency of patients is relatively sparse, and there are many missing values in the normal time-series arrangement, which leads to poor and unstable prediction results. The proposed algorithm can predict stably and efficiently even when there are many vacant values.

For the multitarget classification task of predicting whether a patient‘s phenotype occurs, the average accuracy, area under the curve, and precision-recall area under the curve (PRAUC) of each target are used as evaluation metrics. PRAUC is particularly suitable for imbalanced classification tasks, as it focuses on the model’s performance on the minority class—a common scenario in clinical phenotype prediction where positive cases are often rare. The prediction performance of each model is summarized in Tables 5-7.

**Table 5.** Mean accuracy results for multiple phenotype predictions using the proposed method and advanced time-series methods across 4 datasets.

Data and method	6 hours	12 hours	24 hours	36 hours	48 hours
MIMIC-III^a^					
FCN^b^	0.83 (0.04)	0.82 (0.01)	0.83 (0.02)	0.82 (0.01)	0.83 (0.04)
TCN^c^	0.84 (0.02)	0.83 (0.02)	0.83 (0.01)	0.84 (0.03)	0.84 (0.03)
N-BEATS^d^	0.81 (0.04)	0.80 (0.02)	0.81 (0.01)	0.81 (0.04)	0.83 (0.01)
Crossformer	0.84 (0.02)	0.83 (0.02)	0.84 (0.03)	0.84 (0.03)	0.83 (0.01)
DSformer	0.73 (0.06)	0.69 (0.05)	0.74 (0.06)	0.74 (0.02)	0.73 (0.04)
T-LSTM^e^	0.81 (0.04)	0.76 (0.01)	0.77 (0.06)	0.79 (0.02)	0.76 (0.03)
GRU-D^f^	0.69 (0.03)	0.73 (0.02)	0.70 (0.07)	0.66 (0.02)	0.63 (0.03)
mTAND^g^	0.82 (0.00)	0.83 (0.00)	0.83 (0.01)	0.82 (0.01)	0.82 (0.01)
ContiFormer	0.76 (0.03)	0.71 (0.01)	0.70 (0.05)	0.71 (0.04)	0.72 (0.07)
Our work	0.85 (0.04)	0.84 (0.03)	0.84 (0.04)	0.85 (0.04)	0.84 (0.05)
MIMIC-IV^h^ *ICD-9^i^*					
FCN	0.74 (0.05)	0.76 (0.03)	0.79 (0.01)	0.80 (0.01)	0.80 (0.02)
TCN	0.76 (0.02)	0.76 (0.01)	0.77 (0.02)	0.77 (0.02)	0.79 (0.01)
N-BEATS	0.76 (0.03)	0.78 (0.01)	0.80 (0.03)	0.80 (0.01)	0.75 (0.05)
Crossformer	0.77 (0.02)	0.77 (0.02)	0.81 (0.01)	0.80 (0.02)	0.80 (0.03)
DSformer	0.70 (0.01)	0.71 (0.01)	0.70 (0.04)	0.70 (0.02)	0.68 (0.02)
T-LSTM	0.75 (0.01)	0.75 (0.03)	0.71 (0.05)	0.75 (0.02)	0.70 (0.04)
GRU-D	0.66 (0.03)	0.64 (0.06)	0.62 (0.03)	0.63 (0.03)	0.64 (0.02)
mTAND	0.78 (0.00)	0.77 (0.01)	0.78 (0.00)	0.78 (0.01)	0.77 (0.01)
ContiFormer	0.73 (0.03)	0.72 (0.03)	0.68 (0.02)	0.67 (0.05)	0.72 (0.03)
Our work	0.78 (0.03)	0.81 (0.01)	0.82 (0.04)	0.83 (0.03)	0.83 (0.02)
MIMIC-IV *ICD-10^j^*					
FCN	0.70 (0.02)	0.72 (0.03)	0.71 (0.01)	0.70 (0.04)	0.71 (0.04)
TCN	0.70 (0.03)	0.70 (0.03)	0.69 (0.02)	0.71 (0.02)	0.71 (0.04)
N-BEATS	0.73 (0.01)	0.72 (0.05)	0.73 (0.04)	0.73 (0.04)	0.73 (0.01)
Crossformer	0.75 (0.02)	0.73 (0.02)	0.72 (0.05)	0.73 (0.01)	0.75 (0.03)
DSformer	0.69 (0.04)	0.69 (0.06)	0.69 (0.05)	0.68 (0.05)	0.70 (0.04)
T-LSTM	0.73 (0.02)	0.71 (0.01)	0.73 (0.02)	0.73 (0.02)	0.72 (0.02)
GRU-D	0.69 (0.02)	0.68 (0.02)	0.62 (0.03)	0.66 (0.01)	0.65 (0.02)
mTAND	0.76 (0.00)	0.76 (0.00)	0.75 (0.00)	0.75 (0.00)	0.76 (0.00)
ContiFormer	0.64 (0.02)	0.70 (0.03)	0.68 (0.03)	0.67 (0.03)	0.66 (0.03)
Our work	0.76 (0.02)	0.75 (0.02)	0.76 (0.01)	0.75 (0.03)	0.77 (0.01)
Private dataset					
FCN	0.88 (0.01)	0.87 (0.01)	0.85 (0.02)	0.86 (0.02)	0.88 (0.02)
TCN	0.87 (0.03)	0.86 (0.01)	0.87 (0.04)	0.87 (0.02)	0.87 (0.03)
N-BEATS	0.88 (0.04)	0.88 (0.05)	0.86 (0.06)	0.86 (0.03)	0.86 (0.05)
Crossformer	0.85 (0.01)	0.87 (0.01)	0.88 (0.04)	0.88 (0.02)	0.87 (0.03)
DSformer	0.80 (0.03)	0.80 (0.03)	0.81 (0.02)	0.79 (0.01)	0.80 (0.04)
T-LSTM	0.81 (0.05)	0.83 (0.06)	0.87 (0.02)	0.82 (0.06)	0.83 (0.06)
GRU-D	0.63 (0.05)	0.66 (0.05)	0.63 (0.02)	0.52 (0.07)	0.57 (0.10)
mTAND	0.81 (0.03)	0.81 (0.04)	0.82 (0.03)	0.82 (0.04)	0.82 (0.03)
ContiFormer	0.70 (0.07)	0.74 (0.03)	0.75 (0.02)	0.79 (0.06)	0.68 (0.06)
Our work	0.89 (0.02)	0.89 (0.04)	0.89 (0.02)	0.89 (0.05)	0.91 (0.02)

a MIMIC-III: Medical Information Mart for Intensive Care III.

b FCN: fully convolutional network.

c TCN: temporal convolutional network.

d N-BEATS: neural basis expansion analysis for interpretable time series forecasting.

e T-LSTM: time-aware long short-term memory.

f GRU-D: gated recurrent unit with decay.

g mTAND: multitime attention networks for irregularly sampled time series.

h MIMIC-IV: Medical Information Mart for Intensive Care IV.

i *ICD-9*: *International Classification of Diseases, 9th Revision*.

j *ICD-10*: *International Classification of Diseases, 10th Revision*.

**Table 6.** Mean area under the curve results for multiple phenotypes prediction by the proposed method and advanced time-series methods on four datasets.

Data and method	6 hours	12 hours	24 hours	36 hours	48 hours
MIMIC-III^a^					
FCN^b^	0.63 (0.02)	0.64 (0.01)	0.63 (0.00)	0.64 (0.02)	0.65 (0.01)
TCN^c^	0.70 (0.01)	0.69 (0.00)	0.69 (0.02)	0.70 (0.02)	0.71 (0.03)
N-BEATS^d^	0.65 (0.00)	0.67 (0.01)	0.68 (0.02)	0.67 (0.03)	0.68 (0.01)
Crossformer	0.68 (0.01)	0.70 (0.00)	0.70 (0.01)	0.69 (0.03)	0.70 (0.01)
DSformer	0.58 (0.03)	0.59 (0.03)	0.61 (0.02)	0.61 (0.04)	0.60 (0.02)
T-LSTM^e^	0.60 (0.03)	0.64 (0.00)	0.62 (0.04)	0.61 (0.04)	0.62 (0.04)
GRU-D^f^	0.60 (0.00)	0.63 (0.01)	0.59 (0.02)	0.63 (0.02)	0.65 (0.01)
mTAND^g^	0.64 (0.01)	0.66 (0.00)	0.68 (0.00)	0.70 (0.00)	0.70 (0.00)
ContiFormer	0.60 (0.01)	0.66 (0.00)	0.66 (0.01)	0.68 (0.00)	0.62 (0.08)
Our work	0.70 (0.04)	0.71 (0.02)	0.73 (0.02)	0.74 (0.04)	0.75 (0.04)
MIMIC-IV^h^ *ICD-9^i^*					
FCN	0.65 (0.04)	0.65 (0.02)	0.65 (0.01)	0.66 (0.00)	0.67 (0.00)
TCN	0.66 (0.01)	0.68 (0.01)	0.68 (0.02)	0.70 (0.01)	0.70 (0.02)
N-BEATS	0.68 (0.00)	0.66 (0.01)	0.68 (0.03)	0.68 (0.01)	0.70 (0.01)
Crossformer	0.67 (0.02)	0.70 (0.00)	0.70 (0.01)	0.70 (0.03)	0.71 (0.02)
DSformer	0.60 (0.00)	0.59 (0.01)	0.60 (0.01)	0.62 (0.02)	0.62 (0.02)
T-LSTM	0.62 (0.02)	0.61 (0.03)	0.62 (0.03)	0.62 (0.02)	0.62 (0.04)
GRU-D	0.64 (0.00)	0.65 (0.01)	0.64 (0.01)	0.65 (0.00)	0.63 (0.01)
mTAND	0.67 (0.00)	0.67 (0.01)	0.68 (0.00)	0.69 (0.00)	0.68 (0.00)
ContiFormer	0.66 (0.00)	0.66 (0.02)	0.66 (0.01)	0.66 (0.00)	0.67 (0.00)
Our work	0.72 (0.02)	0.72 (0.00)	0.72 (0.01)	0.74 (0.03)	0.74 (0.02)
MIMIC-IV *ICD-10^j^*					
FCN	0.66 (0.02)	0.66 (0.02)	0.64 (0.03)	0.63 (0.03)	0.65 (0.00)
TCN	0.67 (0.01)	0.67 (0.01)	0.69 (0.00)	0.68 (0.01)	0.65 (0.03)
N-BEATS	0.66 (0.00)	0.67 (0.01)	0.68 (0.02)	0.71 (0.01)	0.72 (0.01)
Crossformer	0.66 (0.03)	0.68 (0.01)	0.69 (0.01)	0.71 (0.03)	0.71 (0.02)
DSformer	0.65 (0.02)	0.64 (0.02)	0.63 (0.01)	0.63 (0.02)	0.64 (0.03)
T-LSTM	0.61 (0.00)	0.63 (0.01)	0.62 (0.03)	0.62 (0.03)	0.63 (0.06)
GRU-D	0.61 (0.02)	0.62 (0.02)	0.63 (0.00)	0.65 (0.01)	0.65 (0.02)
mTAND	0.65 (0.00)	0.66 (0.00)	0.67 (0.00)	0.67 (0.00)	0.67 (0.00)
ContiFormer	0.64 (0.01)	0.65 (0.00)	0.66 (0.01)	0.66 (0.00)	0.66 (0.00)
Our work	0.70 (0.03)	0.72 (0.00)	0.72 (0.01)	0.75 (0.02)	0.74 (0.01)
Private dataset					
FCN	0.65 (0.00)	0.68 (0.01)	0.64 (0.03)	0.65 (0.02)	0.65 (0.00)
TCN	0.63 (0.01)	0.66 (0.02)	0.68 (0.01)	0.68 (0.01)	0.66 (0.01)
N-BEATS	0.65 (0.01)	0.66 (0.00)	0.67 (0.02)	0.71 (0.01)	0.70 (0.00)
Crossformer	0.67 (0.02)	0.69 (0.01)	0.70 (0.04)	0.70 (0.02)	0.71 (0.01)
DSformer	0.67 (0.04)	0.69 (0.02)	0.67 (0.01)	0.63 (0.02)	0.67 (0.03)
T-LSTM	0.64 (0.04)	0.62 (0.05)	0.59 (0.00)	0.63 (0.05)	0.63 (0.07)
GRU-D	0.66 (0.02)	0.68 (0.01)	0.64 (0.03)	0.66 (0.03)	0.65 (0.05)
mTAND	0.66 (0.03)	0.68 (0.00)	0.65 (0.05)	0.66 (0.05)	0.71 (0.01)
ContiFormer	0.68 (0.01)	0.68 (0.02)	0.71 (0.01)	0.64 (0.05)	0.70 (0.01)
Our work	0.72 (0.01)	0.72 (0.03)	0.75 (0.03)	0.74 (0.00)	0.75 (0.01)

a MIMIC-III: Medical Information Mart for Intensive Care III.

b FCN: fully convolutional network.

c TCN: temporal convolutional network.

d N-BEATS: neural basis expansion analysis for interpretable time series forecasting.

e T-LSTM: time-aware long short-term memory.

f GRU-D: gated recurrent unit with decay.

g mTAND: multitime attention networks for irregularly sampled time series.

h MIMIC-IV: Medical Information Mart for Intensive Care IV.

i *ICD-9*: *International Classification of Diseases, 9th Revision*.

j *ICD-10*: *International Classification of Diseases, 10th Revision*.

**Table 7.** Mean precision-recall area under the curve results for multiple phenotypes prediction by the proposed method and advanced time-series methods on 4 datasets.

Data and method	6 hours	12 hours	24 hours	36 hours	48 hours
MIMIC-III^a^					
FCN^b^	0.30 (0.01)	0.35 (0.00)	0.33 (0.01)	0.33 (0.02)	0.35 (0.00)
TCN^c^	0.41 (0.01)	0.43 (0.02)	0.40 (0.01)	0.43 (0.01)	0.45 (0.02)
N-BEATS^d^	0.34 (0.01)	0.36 (0.00)	0.39 (0.00)	0.37 (0.02)	0.36 (0.00)
Crossformer	0.37 (0.00)	0.46 (0.01)	0.44 (0.01)	0.40 (0.01)	0.42 (0.02)
DSformer	0.33 (0.01)	0.31 (0.01)	0.32 (0.01)	0.30 (0.00)	0.39 (0.01)
T-LSTM^e^	0.31 (0.02)	0.34 (0.01)	0.34 (0.02)	0.34 (0.02)	0.33 (0.02)
GRU-D^f^	0.37 (0.01)	0.34 (0.01)	0.32 (0.01)	0.31 (0.01)	0.34 (0.00)
mTAND^g^	0.39 (0.00)	0.37 (0.01)	0.37 (0.01)	0.41 (0.00)	0.42 (0.01)
ContiFormer	0.31 (0.00)	0.32 (0.00)	0.33 (0.00)	0.39 (0.01)	0.35 (0.04)
Our work	0.49 (0.02)	0.51 (0.01)	0.51 (0.01)	0.52 (0.02)	0.55 (0.02)
MIMIC-IV^h^ *ICD-9^i^*					
FCN	0.36 (0.02)	0.38 (0.01)	0.40 (0.00)	0.40 (0.02)	0.42 (0.02)
TCN	0.37 (0.02)	0.45 (0.02)	0.41 (0.01)	0.46 (0.02)	0.48 (0.01)
N-BEATS	0.42 (0.01)	0.41 (0.01)	0.43 (0.02)	0.42 (0.00)	0.45 (0.00)
Crossformer	0.40 (0.01)	0.45 (0.01)	0.47 (0.02)	0.48 (0.02)	0.54 (0.01)
DSformer	0.35 (0.01)	0.36 (0.00)	0.35 (0.00)	0.37 (0.01)	0.36 (0.00)
T-LSTM	0.36 (0.00)	0.37 (0.02)	0.39 (0.01)	0.36 (0.01)	0.36 (0.02)
GRU-D	0.38 (0.01)	0.38 (0.02)	0.37 (0.01)	0.39 (0.01)	0.35 (0.02)
mTAND	0.38 (0.00)	0.38 (0.01)	0.42 (0.02)	0.44 (0.02)	0.44 (0.02)
ContiFormer	0.38 (0.00)	0.40 (0.01)	0.41 (0.00)	0.42 (0.01)	0.41 (0.01)
Our work	0.55 (0.01)	0.57 (0.02)	0.61 (0.02)	0.61 (0.02)	0.59 (0.01)
MIMIC-IV^h^ *ICD-10^j^*					
FCN	0.43 (0.01)	0.40 (0.01)	0.40 (0.02)	0.41 (0.01)	0.41 (0.02)
TCN	0.41 (0.02)	0.45 (0.02)	0.45 (0.01)	0.42 (0.02)	0.39 (0.01)
N-BEATS	0.41 (0.00)	0.43 (0.02)	0.44 (0.01)	0.46 (0.02)	0.48 (0.02)
Crossformer	0.42 (0.01)	0.43 (0.00)	0.45 (0.02)	0.48 (0.02)	0.46 (0.02)
DSformer	0.40 (0.01)	0.39 (0.01)	0.36 (0.02)	0.39 (0.02)	0.40 (0.01)
T-LSTM	0.40 (0.01)	0.39 (0.00)	0.35 (0.02)	0.38 (0.01)	0.37 (0.02)
GRU-D	0.41 (0.01)	0.39 (0.01)	0.38 (0.01)	0.39 (0.02)	0.41 (0.00)
mTAND	0.42 (0.02)	0.41 (0.02)	0.43 (0.01)	0.41 (0.02)	0.43 (0.01)
ContiFormer	0.42 (0.02)	0.40 (0.01)	0.44 (0.02)	0.42 (0.01)	0.40 (0.02)
Our work	0.53 (0.02)	0.59 (0.02)	0.56 (0.02)	0.61 (0.02)	0.60 (0.01)
Private dataset					
FCN	0.36 (0.02)	0.37 (0.00)	0.38 (0.02)	0.36 (0.01)	0.34 (0.01)
TCN	0.34 (0.01)	0.39 (0.01)	0.39 (0.01)	0.39 (0.02)	0.34 (0.02)
N-BEATS	0.36 (0.02)	0.39 (0.00)	0.38 (0.01)	0.42 (0.02)	0.40 (0.01)
Crossformer	0.36 (0.01)	0.39 (0.01)	0.40 (0.02)	0.41 (0.01)	0.43 (0.02)
DSformer	0.38 (0.02)	0.40 (0.01)	0.38 (0.01)	0.36 (0.01)	0.32 (0.04)
T-LSTM	0.41 (0.02)	0.37 (0.02)	0.33 (0.00)	0.34 (0.03)	0.34 (0.04)
GRU-D	0.44 (0.01)	0.40 (0.01)	0.39 (0.01)	0.39 (0.02)	0.38 (0.03)
mTAND	0.43 (0.00)	0.41 (0.01)	0.35 (0.02)	0.38 (0.01)	0.40 (0.01)
ContiFormer	0.45 (0.02)	0.41 (0.01)	0.48 (0.01)	0.42 (0.02)	0.43 (0.02)
Our work	0.51 (0.01)	0.53 (0.02)	0.55 (0.02)	0.53 (0.01)	0.56 (0.02)

a MIMIC-III: Medical Information Mart for Intensive Care III.

b FCN: fully convolutional network.

c TCN: temporal convolutional network.

d N-BEATS: neural basis expansion analysis for interpretable time series forecasting.

e T-LSTM: time-aware long short-term memory.

f GRU-D: gated recurrent unit with decay.

g mTAND: multitime attention networks for irregularly sampled time series.

h MIMIC-IV: Medical Information Mart for Intensive Care IV.

i *ICD-9*: *International Classification of Diseases, 9th Revision*.

j ICD-10: *International Classification of Diseases, 10th Revision*.

From the Tables5  6, it can be seen that the proposed model has achieved superior results in different datasets and different time periods, which fully reflects the advantages of the proposed algorithm in the face of complex downstream tasks. As shown in Table 7, the proposed method consistently achieves the highest PRAUC across most datasets and time windows, indicating its robustness in handling severe class imbalance. Meanwhile, the proposed model demonstrates accuracy in predicting multiple clinical phenotypes on a larger-scale dataset. The detailed comparison results are provided in Multimedia Appendix 1. Comparing different time-series analysis algorithms, the sampling-and-filling method does cause the loss of fine-grained information. However, the data input structure of the graph network proposed in this paper can meet the required input of the model while retaining the fine-grained information in the time-series data to the greatest extent. At the same time, the experimental results show that the simpler the model structure may produce better results when computing time-series data with sampling and filling.

### Regression Prediction Experiment

In the experimental architecture of this paper, predicting the length of hospital stay of a patient is set as a regression prediction analysis task to explore the prediction accuracy of the model in the face of different types of downstream tasks. At the same time, the model can provide multidimensional information required by clinical practice. In this paper, the normalized length of hospital stay of patients is set as the prediction target, and the mean absolute error (MAE) value between the true value and the predicted value is used as the standard to evaluate the quality of the model. The comparison of regression predictions is shown in Table 8. For each dataset, the corresponding minimum and maximum values were used for scaling. Detailed dataset-specific normalization parameters are provided in Multimedia Appendix 3.

**Table 8.** Mean absolute error results for length of hospital stay prediction using the proposed method and advanced time-series methods across 4 datasets. All mean absolute error values are reported on a normalized scale, where stay time was min-max scaled to (0-1).

Data and method	6 hours	12 hours	24 hours	36 hours	48 hours
MIMIC-III^a^					
FCN^b^	0.057 (0.004)	0.049 (0.001)	0.049 (0.003)	0.051 (0.004)	0.047 (0.003)
TCN^c^	0.048 (0.006)	0.057 (0.002)	0.058 (0.002)	0.046 (0.003)	0.167 (0.081)
N-BEATS^d^	0.051 (0.002)	0.058 (0.004)	0.049 (0.005)	0.043 (0.002)	0.049 (0.002)
Crossformer	0.059 (0.009)	0.051 (0.003)	0.048 (0.002)	0.056 (0.003)	0.053 (0.007)
DSformer	0.449 (0.047)	0.155 (0.032)	0.478 (0.026)	0.257 (0.042)	0.104 (0.025)
T-LSTM^e^	0.125 (0.018)	0.084 (0.014)	0.084 (0.012)	0.102 (0.003)	0.098 (0.020)
GRU-D^f^	0.071 (0.002)	0.074 (0.003)	0.066 (0.001)	0.070 (0.002)	0.064 (0.004)
mTAND^g^	0.060 (0.003)	0.051 (0.005)	0.052 (0.006)	0.055 (0.004)	0.049 (0.003)
ContiFormer	0.083 (0.002)	0.079 (0.009)	0.076 (0.012)	0.057 (0.012)	0.078 (0.020)
Our work	0.045 (0.002)	0.047 (0.003)	0.044 (0.001)	0.047 (0.003)	0.042 (0.004)
MIMIC-IV^h^ *ICD-9^i^*					
FCN	0.067 (0.004)	0.076 (0.003)	0.075 (0.005)	0.083 (0.003)	0.079 (0.002)
TCN	0.091 (0.006)	0.086 (0.005)	0.087 (0.002)	0.090 (0.002)	0.084 (0.007)
N-BEATS	0.080 (0.003)	0.072 (0.007)	0.069 (0.004)	0.076 (0.001)	0.070 (0.003)
Crossformer	0.059 (0.001)	0.074 (0.002)	0.073 (0.001)	0.079 (0.005)	0.069 (0.005)
DSformer	0.247 (0.041)	0.256 (0.053)	0.226 (0.037)	0.110 (0.011)	0.117 (0.015)
T-LSTM	0.071 (0.007)	0.091 (0.013)	0.104 (0.016)	0.093 (0.007)	0.079 (0.009)
GRU-D	0.073 (0.002)	0.075 (0.002)	0.074 (0.002)	0.070 (0.001)	0.071 (0.001)
mTAND	0.065 (0.002)	0.069 (0.005)	0.070 (0.009)	0.074 (0.008)	0.075 (0.005)
ContiFormer	0.075 (0.003)	0.074 (0.003)	0.088 (0.023)	0.079 (0.011)	0.071 (0.011)
Our work	0.057 (0.003)	0.046 (0.005)	0.057 (0.003)	0.057 (0.002)	0.056 (0.003)
MIMIC-IV *ICD-10^j^*					
FCN	0.070 (0.004)	0.062 (0.007)	0.067 (0.004)	0.085 (0.004)	0.086 (0.007)
TCN	0.082 (0.006)	0.083 (0.006)	0.085 (0.011)	0.072 (0.005)	0.072 (0.009)
N-BEATS	0.064 (0.009)	0.060 (0.002)	0.069 (0.003)	0.078 (0.006)	0.083 (0.006)
Crossformer	0.052 (0.000)	0.063 (0.010)	0.077 (0.006)	0.083 (0.007)	0.070 (0.003)
DSformer	0.323 (0.015)	0.184 (0.023)	0.080 (0.002)	0.086 (0.005)	0.078 (0.004)
T-LSTM	0.107 (0.017)	0.097 (0.014)	0.116 (0.016)	0.119 (0.014)	0.110 (0.008)
GRU-D	0.048 (0.003)	0.059 (0.002)	0.048 (0.004)	0.067 (0.003)	0.058 (0.003)
mTAND	0.074 (0.006)	0.068 (0.005)	0.070 (0.005)	0.078 (0.006)	0.076 (0.003)
ContiFormer	0.090 (0.025)	0.080 (0.003)	0.158 (0.042)	0.093 (0.017)	0.086 (0.006)
Our work	0.051 (0.002)	0.057 (0.004)	0.062 (0.002)	0.061 (0.003)	0.056 (0.004)
Private dataset					
FCN	0.057 (0.010)	0.044 (0.000)	0.049 (0.004)	0.043 (0.005)	0.041 (0.002)
TCN	0.057 (0.004)	0.049 (0.000)	0.055 (0.002)	0.050 (0.003)	0.048 (0.004)
N-BEATS	0.056 (0.009)	0.044 (0.002)	0.048 (0.005)	0.047 (0.005)	0.049 (0.005)
Crossformer	0.049 (0.003)	0.050 (0.003)	0.049 (0.001)	0.050 (0.007)	0.053 (0.008)
DSformer	0.381 (0.043)	0.397 (0.094)	0.195 (0.026)	0.227 (0.053)	0.396 (0.082)
T-LSTM	0.137 (0.020)	0.168 (0.015)	0.134 (0.005)	0.161 (0.002)	0.164 (0.009)
GRU-D	0.061 (0.003)	0.060 (0.005)	0.062 (0.001)	0.057 (0.005)	0.055 (0.002)
mTAND	0.060 (0.005)	0.050 (0.007)	0.053 (0.007)	0.056 (0.009)	0.052 (0.006)
ContiFormer	0.139 (0.010)	0.156 (0.011)	0.132 (0.006)	0.130 (0.010)	0.122 (0.050)
Our work	0.038 (0.003)	0.040 (0.004)	0.042 (0.000)	0.039 (0.003)	0.044 (0.004)

a MIMIC-III: Medical Information Mart for Intensive Care III.

b FCN: fully convolutional network.

c TCN: temporal convolutional network.

d N-BEATS: neural basis expansion analysis for interpretable time series forecasting.

e T-LSTM: time-aware long short-term memory.

f GRU-D: gated recurrent unit with decay.

g mTAND: multitime attention networks for irregularly sampled time series.

h MIMIC-IV: Medical Information Mart for Intensive Care IV.

i *ICD-9*: *International Classification of Diseases, 9th Revision*.

j *ICD-10: International Classification of Diseases, 10th Revision.*

From the analysis of the table, it can be found that the prediction accuracy of the model proposed in this paper is in the leading position in most datasets and time periods and is slightly inferior to the best prediction results in a small number of time periods. This finding fully reflects the stability of the model proposed in this paper in the face of different downstream tasks. Meanwhile, the proposed model demonstrates accuracy in predicting the length of hospital stay on a larger-scale dataset. The detailed comparison results are provided in Multimedia Appendix 1. Compared with other models, it can be found that some time-series analysis models have good accuracy in classification experiments, but have large deviation values in regression prediction tasks. In this paper, the prediction indicators of different time periods of each dataset are averaged, and the regression prediction and classification prediction are projected into the same set of score space to verify the stability of the model in the face of multitask learning.

### Ablation Experiment

In the ablation experiment of the model, the point feature space and edge weight space proposed in this paper are experimented on using the above 3 regression and prediction analysis tasks (death, phenotype, and stay time) on 4 datasets with different time lengths, and the contribution of feature vectors extracted from different feature spaces to the overall model is explored. Meanwhile, this paper also analyzed the connection results of 2 feature spaces under the learnable head and the direct connection results of 2 feature spaces. Results from top to bottom, the accuracy for predicting death, the average accuracy for predicting the occurrence of different representations of patients, and the MAE for predicting the length of hospital stay were arranged. The values are given in Table 9.

**Table 9.** Results from the ablation study evaluating the proposed method across 3 prediction dimensions across 4 datasets.

Data, method, and result	6 hours	12 hours	24 hours	36 hours	48 hours
MIMIC-III^a^					
Point					
Death accuracy	0.7200	0.7575	0.7175	0.7000	0.6875
Phenotype accuracy	0.801	0.813	0.805	0.812	0.809
Stay MAE^b^	0.1078	0.1351	0.1383	0.1562	0.1513
Edge					
Death accuracy	0.7125	0.7350	0.7075	0.7425	0.7500
Phenotype accuracy	0.821	0.825	0.821	0.818	0.820
Stay MAE	0.0535	0.0574	0.0511	0.0552	0.0521
Point + Edge (DC)^c^					
Death accuracy	0.7375	0.7525	0.7225	0.7500	0.7675
Phenotype accuracy	0.840	0.838	0.830	0.835	0.832
Stay MAE	0.0512	0.0534	0.0503	0.0537	0.0520
Point + Edge^d^					
Death accuracy	0.7525	0.7775	0.7375	0.7750	0.8100
Phenotype accuracy	0.847	0.838	0.840	0.851	0.842
Stay MAE	0.0447	0.0459	0.0450	0.0451	0.0445
MIMIC-IV^e^ *ICD-9^f^*					
Point					
Death accuracy	0.6075	0.8075	0.8600	0.8050	0.7825
Phenotype accuracy	0.774	0.771	0.783	0.795	0.804
Stay MAE	0.6481	0.6124	0.6235	0.6265	0.6226
Edge					
Death accuracy	0.8625	0.8225	0.8525	0.8050	0.8025
Phenotype accuracy	0.789	0.791	0.829	0.820	0.831
Stay MAE	0.0745	0.0699	0.0717	0.0642	0.0639
Point + Edge (DC)					
Death accuracy	0.8775	0.8450	0.8825	0.8000	0.8125
Phenotype accuracy	0.790	0.795	0.829	0.824	0.830
Stay MAE	0.0657	0.0546	0.0641	0.0619	0.0606
Point + Edge					
Death accuracy	0.8875	0.8575	0.9000	0.8575	0.8175
Phenotype accuracy	0.796	0.803	0.832	0.835	0.836
Stay MAE	0.0577	0.0494	0.0585	0.0594	0.0559
MIMIC-IV *ICD-10^g^*					
Point					
Death accuracy	0.7575	0.8200	0.8425	0.8800	0.8600
Phenotype accuracy	0.701	0.714	0.705	0.710	0.708
Stay MAE	0.6506	0.6457	0.6479	0.6381	0.6535
Edge					
Death accuracy	0.7375	0.8625	0.8450	0.8500	0.8275
Phenotype accuracy	0.731	0.717	0.727	0.721	0.734
Stay MAE	0.0693	0.0599	0.0686	0.0770	0.0823
Point + Edge (DC)					
Death accuracy	0.8125	0.8700	0.8525	0.8925	0.8500
Phenotype accuracy	0.743	0.725	0.738	0.729	0.746
Stay MAE	0.0603	0.0609	0.0634	0.0727	0.0691
Point + Edge					
Death accuracy	0.8475	0.8875	0.8775	0.9200	0.8650
Phenotype accuracy	0.754	0.737	0.746	0.749	0.757
Stay MAE	0.0514	0.0566	0.0609	0.0646	0.0573
Private dataset					
Point					
Death accuracy	0.6075	0.5575	0.5950	0.6300	0.5825
Phenotype accuracy	0.784	0.791	0.797	0.811	0.799
Stay MAE	0.6608	0.6633	0.6703	0.6705	0.6580
Edge					
Death accuracy	0.5875	0.5175	0.7175	0.7025	0.6050
Phenotype accuracy	0.827	0.843	0.855	0.841	0.859
Stay MAE	0.0355	0.0464	0.0435	0.0447	0.0435
Point + Edge (DC)					
Death accuracy	0.6375	0.7250	0.7350	0.7050	0.7075
Phenotype accuracy	0.864	0.856	0.871	0.877	0.883
Stay MAE	0.0402	0.0478	0.0454	0.0459	0.0519
Point + Edge					
Death accuracy	0.6775	0.7375	0.7475	0.7050	0.7250
Phenotype accuracy	0.898	0.899	0.902	0.906	0.905
Stay MAE	0.0355	0.0421	0.0416	0.0382	0.0424

a MIMIC-III: Medical Information Mart for Intensive Care III.

b MAE: mean absolute error.

c Direct connection of point features and edge features (without the learnable head).

d The connection between point features and edge features under the learnable head.

e MIMIC-IV: Medical Information Mart for Intensive Care IV.

f *ICD-9: International Classification of Diseases, 9th Revision.*

g *ICD-10*: *International Classification of Diseases, 10th Revision.*

From the analysis of the table, it can be found that both the point feature space and the edge weight space can achieve certain prediction accuracy in the classification task. However, due to the characteristics of the graph network space and the fact that the GCN network is easy to fall into local graph space, the features extracted from the point feature space lead to unstable prediction results. The lack of measurement time information in the edge weight space also leads to low prediction accuracy of the extracted features. The combination of the two can effectively construct a stable and high-accuracy prediction model. In regression prediction analysis, the information provided by the point feature space is limited, and the results of regression prediction have large deviations. Under the action of the learnable head, it can effectively fuse with the feature vector extracted from the edge weight space. It improves the prediction accuracy and prediction stability of the model. Meanwhile, a single-head attention mechanism network and a single-layer GCN are supplemented to verify the effectiveness of hyperparameter configuration and the construction of the multilevel network framework. The results are shown in Table 10.

**Table 10.** Results from the network parameter ablation evaluating the proposed method across 3 prediction dimensions across 4 datasets.

Data, hours, and method	Death accuracy	Death AUC^a^	Stay MAE^b^	Phenotype accuracy	Phenotype AUC
MIMIC^c^-III					
6 hours					
Trans_1	0.574	0.581	0.0581	0.824	0.651
GCN_1^d^	0.610	0.626	0.1871	0.736	0.629
12 hours					
Trans_1	0.572	0.585	0.0556	0.827	0.661
GCN_1	0.618	0.636	0.1316	0.615	0.608
24 hours					
Trans_1	0.569	0.581	0.0610	0.817	0.676
GCN_1	0.618	0.637	0.1100	0.736	0.634
36 hours					
Trans_1	0.591	0.605	0.0550	0.817	0.682
GCN_1	0.654	0.663	0.1007	0.636	0.628
48 hours					
Trans_1	0.592	0.615	0.0532	0.817	0.687
GCN_1	0.664	0.689	0.0787	0.733	0.641
MIMIC-IV^e^ *ICD-9^f^*					
6 hours					
Trans_1	0.757	0.819	0.0654	0.767	0.656
GCN_1	0.602	0.679	0.1800	0.732	0.653
12 hours					
Trans_1	0.740	0.800	0.0618	0.744	0.666
GCN_1	0.622	0.759	0.1449	0.681	0.619
24 hours					
Trans_1	0.751	0.818	0.0601	0.746	0.665
GCN_1	0.600	0.685	0.1610	0.681	0.635
36 hours					
Trans_1	0.757	0.818	0.0609	0.745	0.689
GCN_1	0.702	0.771	0.2065	0.680	0.613
48 hours					
Trans_1	0.779	0.832	0.0729	0.734	0.676
GCN_1	0.742	0.797	0.1379	0.681	0.606
MIMIC-IV *ICD-10^g^*					
6 Hours					
Trans_1	0.703	0.743	0.0744	0.727	0.654
GCN_1	0.600	0.620	0.1055	0.687	0.613
12 hours					
Trans_1	0.708	0.755	0.0752	0.717	0.663
GCN_1	0.597	0.657	0.2773	0.663	0.615
24 hours					
Trans_1	0.722	0.779	0.0708	0.742	0.663
GCN_1	0.602	0.684	0.1695	0.664	0.608
36 hours					
Trans_1	0.733	0.789	0.0709	0.728	0.664
GCN_1	0.653	0.730	0.2295	0.664	0.616
48 hours					
Trans_1	0.747	0.795	0.0746	0.763	0.672
GCN_1	0.675	0.753	0.1843	0.678	0.642
Private dataset					
6 hours					
Trans_1	0.612	0.624	0.0565	0.839	0.672
GCN_1	0.602	0.612	0.2295	0.787	0.642
12 hours					
Trans_1	0.617	0.646	0.0594	0.859	0.645
GCN_1	0.600	0.570	0.2238	0.793	0.630
24 hours					
Trans_1	0.587	0.625	0.0565	0.873	0.569
GCN_1	0.597	0.636	0.1184	0.792	0.637
36 hours					
Trans_1	0.597	0.634	0.1107	0.887	0.554
GCN_1	0.601	0.588	0.4836	0.792	0.657
48 hours					
Trans_1	0.610	0.652	0.0530	0.870	0.572
GCN_1	0.608	0.614	0.3325	0.737	0.636

a AUC: area under the curve.

b MAE: mean absolute error.

c MIMIC-III: Medical Information Mart for Intensive Care III.

d GCN: graph convolutional network.

e MIMIC-IV: Medical Information Mart for Intensive Care IV.

f *ICD-9: International Classification of Diseases, 9th Revision.*

g *ICD-10: International Classification of Diseases, 10th Revision.*

## Discussion

### Principal Findings

**Figure 3.**
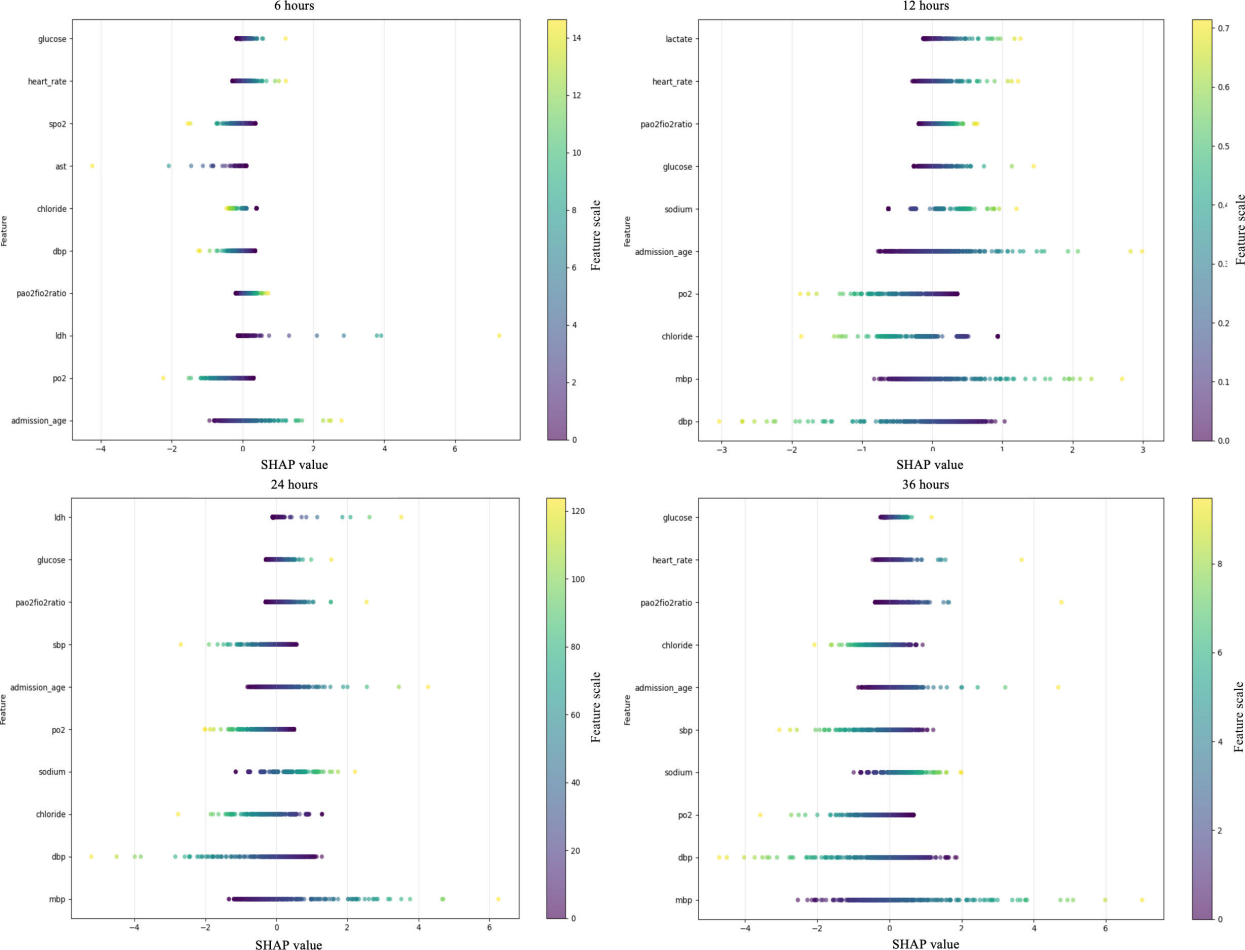
Shapley Additive Explanations (SHAP) analysis figure: variation of feature contributions across different time points.

Aiming at the problem of uneven sampling time intervals and a large number of missing values in medical time-series data, this paper proposes to transform medical time-series data into a graph network structure for input. The feature vectors are extracted by constructing algorithms respectively for the point feature space and the edge weight space in the graph network data structure and are fused to form the feature vector of the graph network space under the action of the learnable head. The fused features are then decoded by the decoder to cope with different downstream tasks. In the exploration of model interpretability, this study extracts each feature at different time points from the trained model. The extracted features are fitted to different target tasks using logistic regression, and Shapley Additive Explanations analysis is used to obtain the changes in the contribution of features to the final target task across various time points. Figure 3 illustrates the variation of feature contributions to mortality prediction at different time scales based on 48-hour data from the MIMIC-IV *ICD-10* dataset. Results for the entire dataset are provided in Multimedia Appendix 4.

As analyzed from Figure 3, heart rate and glucose indicators are important factors affecting patient survival in the early stage of hospitalization. With the passage of time, carbohydrate metabolites and patient age gradually become the dominant factors.

### Comparison to Prior Work

In this paper, the proposed model is tested in 5 different data time ranges in 4 datasets, and it is found that the proposed model has good prediction accuracy and prediction stability in classification and regression prediction tasks. Meanwhile, paired DeLong tests were performed on the mortality probability prediction results of the proposed model and comparative models in the MIMIC-IV *ICD-9* and private datasets. The results showed that the *P* value of the test in MIMIC-IV *ICD-9* was <0.001 and that in private data was <0.05. Additionally, paired *U*-tests were conducted on the MAE values of the regression predictions for length of hospital stay, revealing that *P* values in both MIMIC-IV *ICD-9* and private data were <0.05. The specific value of *P* is provided in Multimedia Appendix 5. These findings fully demonstrate the effectiveness of the proposed model in improving prediction performance. Moreover, this paper conducts ablation experiments on the point feature space model and the edge weight space model and finds that the accuracy and stability of the classification prediction of the model come from the simultaneous contributions of the 2 spaces. The accuracy of regression prediction comes from the adjustment and fusion of the 2 spatial feature vectors by the learnable head.

In comparing our proposed model with existing methods, it is important to acknowledge a confounding factor in the baseline experimental design. Our graph-based model operates directly on raw, irregularly sampled time-series data without any imputation, whereas standard deep learning models, such as a fully convolutional network and temporal convolutional network, are trained on data that have been preprocessed with mean imputation to enforce regular sampling intervals. This design choice conflates 2 distinct sources of potential performance gain: the architectural advantage of the graph network and the representational benefit of avoiding information loss due to imputation. The primary rationale for this comparison was to reflect a realistic clinical deployment scenario, where missing values are prevalent, and imputation remains the default preprocessing step for conventional models. As such, the current benchmark captures the end-to-end practical advantage of a model that inherently accommodates irregular data without the need for imputation. However, we acknowledge that this setup does not isolate the contribution of the graph architecture alone. Despite this limitation, we believe the current comparison remains clinically meaningful, as it reflects the performance gap between models that require imputation and those that do not in real-world settings.

### Limitations

The model proposed in this paper also has the following limitations: (1) since the method proposed in this study fully uses the information in the data to construct nodes and edges and uses both nodes and edges for computation simultaneously, it imposes certain requirements on the training space; and (2) as the number of computational features and the scope of computation time increase, the demand for computational space grows exponentially.

### Future Directions

In the future, this study intends to develop a graph network construction paradigm with a more compact structure, which can be compatible with longer time-series data and more features, thereby enabling the tracking and monitoring of patients’ overall disease progression. While the proposed model demonstrates strong predictive performance on irregularly sampled medical time-series data, its scalability to large-scale clinical databases remains a challenge. As noted in the Limitations subheading, the current graph construction and joint computation over node and edge feature spaces impose significant memory overhead that grows exponentially with the number of features and time points. To facilitate real-world deployment in hospital informatics environments that routinely handle tens of thousands of patients, several optimization strategies can be explored. First, sparse graph representations could be adopted to retain only clinically meaningful edges, reducing the memory footprint. Second, feature or temporal down-sampling techniques may help control the node count while preserving essential information. Third, distributed computing frameworks or graph mini-batching strategies could partition the computation across multiple devices, enabling parallel processing of large patient cohorts. Finally, memory-efficient graph convolution operators, such as those based on neighborhood sampling or low-rank approximations, offer promising avenues for scaling graph networks to larger datasets. In future work, we aim to investigate these directions to enhance the scalability and clinical utility of the model without compromising predictive accuracy.

### Conclusions

Based on the model’s prediction results, analyzing patients’ clinical time-series data from the early time period can reveal the disease progression for many patients. In addition to carefully examining the characteristic representations of patients identified by the model as having higher mortality risk, it is also possible to focus on predicting treatment regimens that are likely to be misapplied to a given patient. Through the similarity of the patient’s graph network to form the edge and through the hierarchical clustering method, a good treatment plan is mined, and the wrong treatment plan is excluded. Overall, this paper proposes a deep learning–based temporal data analysis model for accurately predicting the survival outcomes of critically ill patients, while also providing critical features that require close attention at various time points.

## Supplementary material

10.2196/81145 Multimedia Appendix 1 Model validity verification.

10.2196/81145 Multimedia Appendix 2 Model parameters.

10.2196/81145 Multimedia Appendix 3 Detailed dataset-specific normalization parameters.

10.2196/81145 Multimedia Appendix 4 Shapley Additive Explanations analysis figures.

10.2196/81145 Multimedia Appendix 5 Statistical test results.

## Abbreviations

GCN: graph convolutional network
*ICD-10*: *International Classification of Diseases, 10th Revision*
*ICD-9*: *International Classification of Diseases, 9th Revision*
KL: Kullback-Leibler
MAE: mean absolute error
MIMIC: Medical Information Mart for Intensive Care
PRAUC: precision-recall area under the curve
