# Supplementary material for: Graph Network Feature Space Fusion for Predicting Irregularly Sampled Medical Time-Series Data: Deep Learning Model Development and Validation Study
Source: JMIR Med Inform. 2026 Jul 3;14:e81145. doi: 10.2196/81145 (PMC13331332; doi:10.2196/81145)
Supplement: Multimedia Appendix 1 [file medinform-v14-e81145-s001.docx]

| Data | Hours | Method | death_acc | death_auc | Stay_mae | Phe_acc | Phe_auc |
| --- | --- | --- | --- | --- | --- | --- | --- |
| MIMIC-III | 6 Hours | T-Lstm | 0.82(0.02) | 0.60(0.03) | 0.131(0.009) | 0.80(0.03) | 0.62(0.04) |
|  |  | Grud | 0.70(0.02) | 0.63(0.04) | 0.085(0.007) | 0.71(0.04) | 0.61(0.05) |
|  |  | mTAND | 0.80(0.02) | 0.59(0.02) | 0.058(0.002) | 0.81(0.03) | 0.64(0.02) |
|  |  | ContiFormer | 0.76(0.01) | 0.62(0.03) | 0.076(0.003) | 0.79(0.01) | 0.62(0.03) |
|  |  | Our work | **0.86(0.03)** | **0.75(0.02)** | **0.044(0.004)** | **0.87(0.01)** | **0.71(0.03)** |
|  | 12Hours | T-Lstm | 0.79(0.02) | 0.60(0.04) | 0.080(0.018) | 0.78(0.02) | 0.67(0.03) |
|  |  | Grud | 0.75(0.03) | 0.65(0.02) | 0.075(0.006) | 0.75(0.03) | 0.65(0.02) |
|  |  | mTAND | 0.80(0.04) | 0.64(0.03) | 0.060(0.009) | 0.81(0.04) | 0.65(0.00) |
|  |  | ContiFormer | 0.73(0.03) | 0.64(0.02) | 0.076(0.005) | 0.73(0.04) | 0.67(0.01) |
|  |  | Our work | **0.86(0.02)** | **0.75(0.03)** | **0.046(0.004)** | **0.86(0.01)** | **0.74(0.02)** |
| MIMIC-IV_ICD9 | 6 Hours | T-Lstm | 0.75(0.01) | 0.79(0.00) | 0.079(0.005) | 0.76(0.02) | 0.65(0.01) |
|  |  | Grud | 0.72(0.01) | 0.77(0.02) | 0.079(0.007) | 0.68(0.01) | 0.65(0.00) |
|  |  | mTAND | 0.75(0.00) | 0.80(0.01) | 0.060(0.004) | 0.77(0.01) | 0.67(0.00) |
|  |  | ContiFormer | 0.75(0.01) | 0.70(0.01) | 0.070(0.008) | 0.74(0.02) | 0.67(0.00) |
|  |  | Our work | **0.84(0.02)** | **0.85(0.01)** | **0.055(0.004)** | **0.79(0.01)** | **0.75(0.03)** |
|  | 12Hours | T-Lstm | 0.75(0.00) | 0.78(0.00) | 0.094(0.011) | 0.74(0.02) | 0.64(0.01) |
|  |  | Grud | 0.74(0.02) | 0.78(0.01) | 0.076(0.005) | 0.67(0.02) | 0.66(0.02) |
|  |  | mTAND | 0.76(0.01) | 0.79(0.02) | 0.067(0.004) | 0.74(0.02) | 0.66(0.01) |
|  |  | ContiFormer | 0.74(0.00) | 0.70(0.02) | 0.071(0.002) | 0.75(0.01) | 0.68(0.02) |
|  |  | Our work | **0.85(0.02)** | **0.88(0.01)** | **0.043(0.003)** | **0.81(0.02)** | **0.74(0.01)** |
| MIMIC-IV_ICD10 | 6 Hours | T-Lstm | 0.70(0.02) | 0.71(0.02) | 0.091(0.012) | 0.75(0.01) | 0.69(0.01) |
|  |  | Grud | 0.71(0.00) | 0.74(0.01) | 0.053(0.003) | 0.71(0.03) | 0.70(0.01) |
|  |  | mTAND | 0.71(0.01) | 0.73(0.00) | 0.071(0.003) | **0.77(0.01)** | 0.71(0.00) |
|  |  | ContiFormer | 0.73(0.03) | 0.71(0.03) | 0.088(0.019) | 0.73(0.01) | 0.70(0.01) |
|  |  | Our work | **0.83(0.01)** | **0.77(0.01)** | **0.050(0.004)** | **0.77(0.01)** | **0.73(0.02)** |
|  | 12Hours | T-Lstm | 0.72(0.01) | 0.72(0.01) | 0.090(0.009) | 0.70(0.02) | 0.70(0.01) |
|  |  | Grud | 0.71(0.02) | 0.75(0.01) | 0.061(0.003) | 0.70(0.01) | 0.72(0.02) |
|  |  | mTAND | 0.73(0.01) | 0.76(0.01) | 0.070(0.006) | 0.75(0.02) | 0.74(0.01) |
|  |  | ContiFormer | 0.77(0.01) | 0.74(0.00) | 0.083(0.004) | 0.74(0.02) | 0.72(0.00) |
|  |  | Our work | **0.85(0.02)** | **0.80(0.02)** | **0.056(0.002)** | **0.76(0.01)** | **0.75(0.02)** |
| Private Dataset | 6 Hours | T-Lstm | 0.61(0.00) | 0.65(0.02) | 0.121(0.013) | 0.80(0.03) | 0.74(0.03) |
|  |  | Grud | 0.65(0.01) | 0.66(0.00) | 0.078(0.007) | 0.78(0.03) | 0.76(0.01) |
|  |  | mTAND | 0.69(0.01) | 0.66(0.01) | 0.069(0.002) | 0.82(0.04) | 0.70(0.02) |
|  |  | ContiFormer | 0.69(0.02) | 0.70(0.01) | 0.151(0.008) | 0.81(0.02) | 0.74(0.03) |
|  |  | Our work | **0.71(0.01)** | **0.72(0.01)** | **0.042(0.003)** | 0.89(0.05) | **0.78(0.03)** |
|  | 12Hours | T-Lstm | 0.66(0.01) | 0.67(0.01) | 0.158(0.021) | 0.83(0.06) | 0.72(0.01) |
|  |  | Grud | 0.65(0.02) | 0.61(0.00) | 0.063(0.003) | 0.70(0.07) | 0.74(0.02) |
|  |  | mTAND | 0.69(0.01) | 0.67(0.01) | 0.057(0.004) | 0.79(0.03) | 0.74(0.01) |
|  |  | ContiFormer | 0.73(0.02) | 0.68(0.01) | 0.141(0.017) | 0.75(0.09) | 0.73(0.01) |
|  |  | Our work | **0.76(0.01)** | **0.75(0.00)** | **0.045(0.003)** | 0.88(0.02) | **0.77(0.02)** |
