# Supplementary material for: Graph Network Feature Space Fusion for Predicting Irregularly Sampled Medical Time-Series Data: Deep Learning Model Development and Validation Study
Source: JMIR Med Inform. 2026 Jul 3;14:e81145. doi: 10.2196/81145 (PMC13331332; doi:10.2196/81145)
Supplement: Multimedia Appendix 2 [file medinform-v14-e81145-s002.docx]

| Method_name | Model Parameters |
| --- | --- |
| FCN | Conv1d layer 1 = 42, out_channels = 128, kernel_size = 8  Conv1d layer 2 = in_channels = 128, out_channels = 256, kernel_size = 5  Conv1d layer 3 = in_channels = 256, out_channels = 128, kernel_size = 3  Output layer (classification) = Linear(in_features = 128, out_features = 2)  Output layer (forecasting) = Linear(in_features = 128, out_features = 1)  Dropout rate = 0.2  learning rate = 1e-3  Batch size = 32  Epochs=100 |
| TCN | Dilation rate = 1, 2, 4, 8  Number of residual blocks = 3  Kernel size = 3  Initial learning rate = 3e-4  Batch size =32  Epochs=80 |
| Nbeats | Number of stacks =4  Hidden layer dimensions= [512, 256, 128]  Initial learning rate = 1e-3  Batch size =32  Epochs=140 |
| Crossformer | Segment size = 48  Number of encoder layers = 3  Number of attention heads = 4  d_model (hidden dimension) = 512  Initial learning rate = 2e-4  Batch size =8  Epochs=100 |
| Dsformer | Local attention window size = 32  Global sparse sampling rate = 0.1  Number of encoder layers = 4  Number of decoder layers = 2  d_model = 256  Number of attention heads = 4  Initial learning rate = 2e-4  Batch size =8  Epochs=100 |
| T-Lstm | Hidden dimension = 128  Number of layers = 2  Input dropout rate = 0.2  Hidden dropout rate = 0.2  Input window length = 32  Initial learning rate = 2e-4  Batch size =32  Epochs=200 |
| Grud | Hidden dimension = 128  Number of layers = 1  Initial learning rate = 2e-4  Batch size =32  Epochs=200 |
| mTAND | Number of attention heads = 4  CNN channel dimensions = 64 → 128  LSTM hidden dimension = 128  Initial learning rate = 2e-4  Batch size =32  Epochs=200 |
| ContiFormer | hidden_dim=64  num_heads=2  num_layers=2  Initial learning rate = 2e-4  Batch size =16  Epochs=200 |
| Our work | hiden_layer=64  dropout_rate = 0.5  GCN layer num=2  head_num=2  Attention layer num= 3  Initial learning rate = 2e-4  Batch size =2  Epochs=200 |
