# Supplementary material for: Graph Network Feature Space Fusion for Predicting Irregularly Sampled Medical Time-Series Data: Deep Learning Model Development and Validation Study
Source: JMIR Med Inform. 2026 Jul 3;14:e81145. doi: 10.2196/81145 (PMC13331332; doi:10.2196/81145)
Supplement: Multimedia Appendix 3 [file medinform-v14-e81145-s003.docx]

| Data | Length of Hospital Stay（max） | Length of Hospital Stay（min） |
| --- | --- | --- |
| MIMIC-III | 53.67 | 378.43 |
| MIMIC-IV_ICD9 | 65.25 | 391.83 |
| MIMIC-IV_ICD10 | 56.33 | 630.67 |
| Private Dataset | 92.33 | 717.50 |
