# Supplementary figures and images for: Graph Network Feature Space Fusion for Predicting Irregularly Sampled Medical Time-Series Data: Deep Learning Model Development and Validation Study
Source: JMIR Med Inform. 2026 Jul 3;14:e81145. doi: 10.2196/81145 (PMC13331332; doi:10.2196/81145)

1. Private data


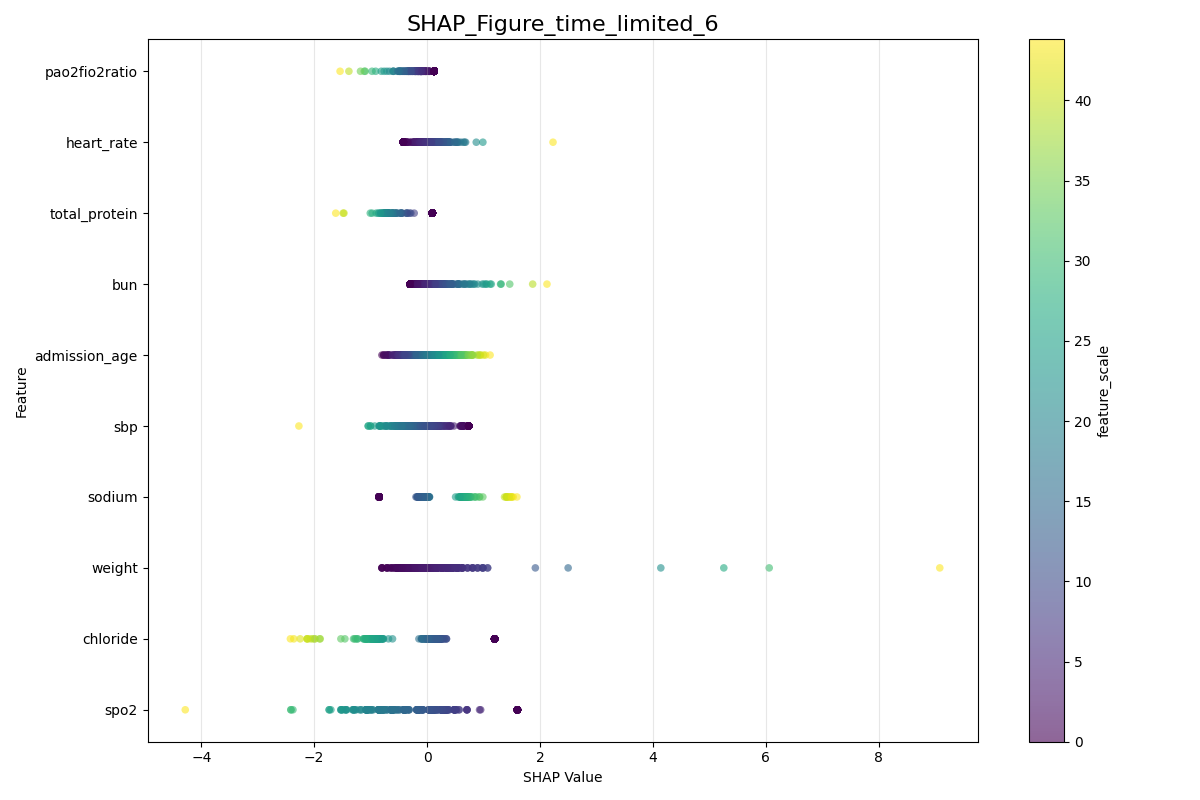


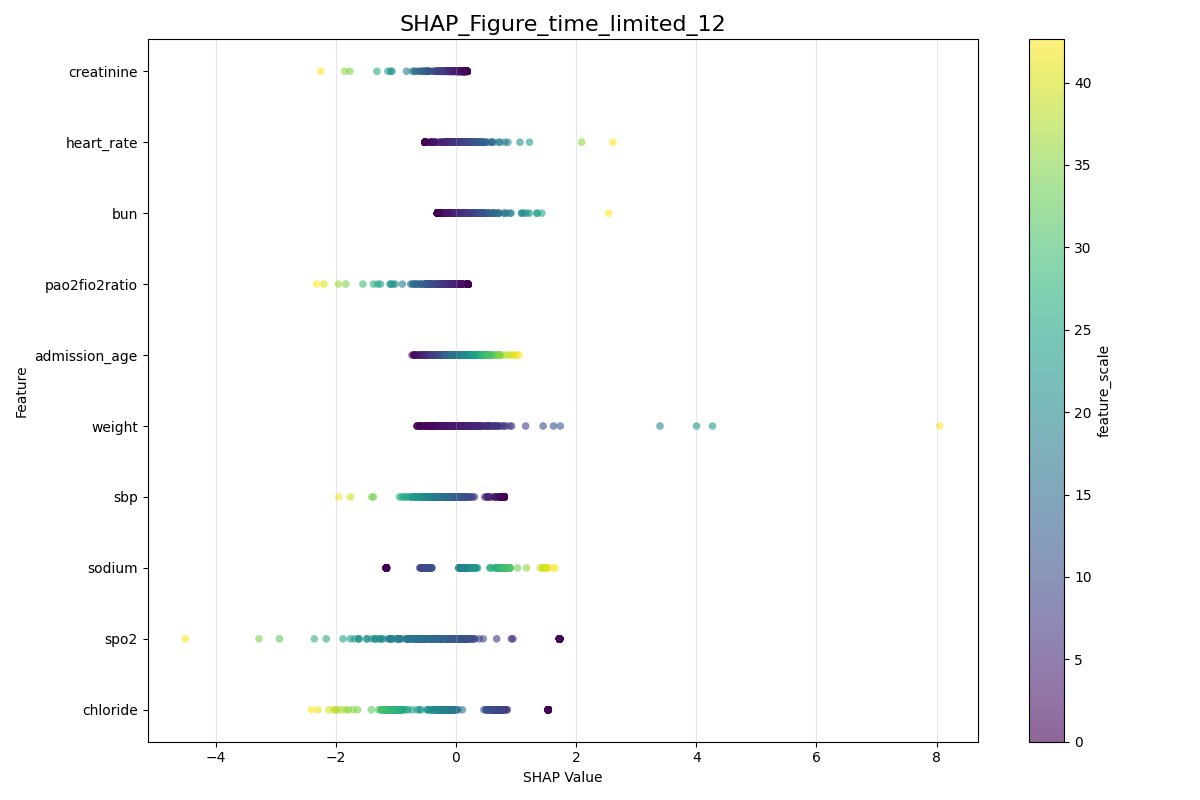


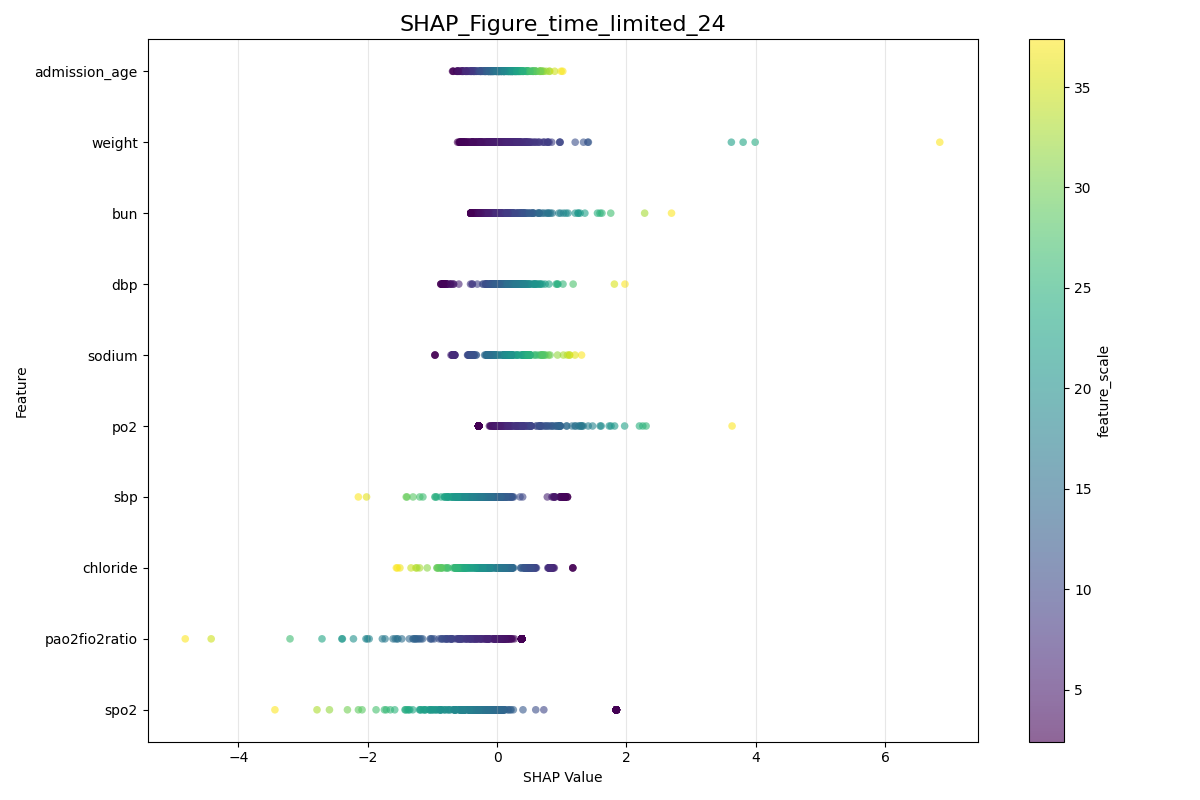


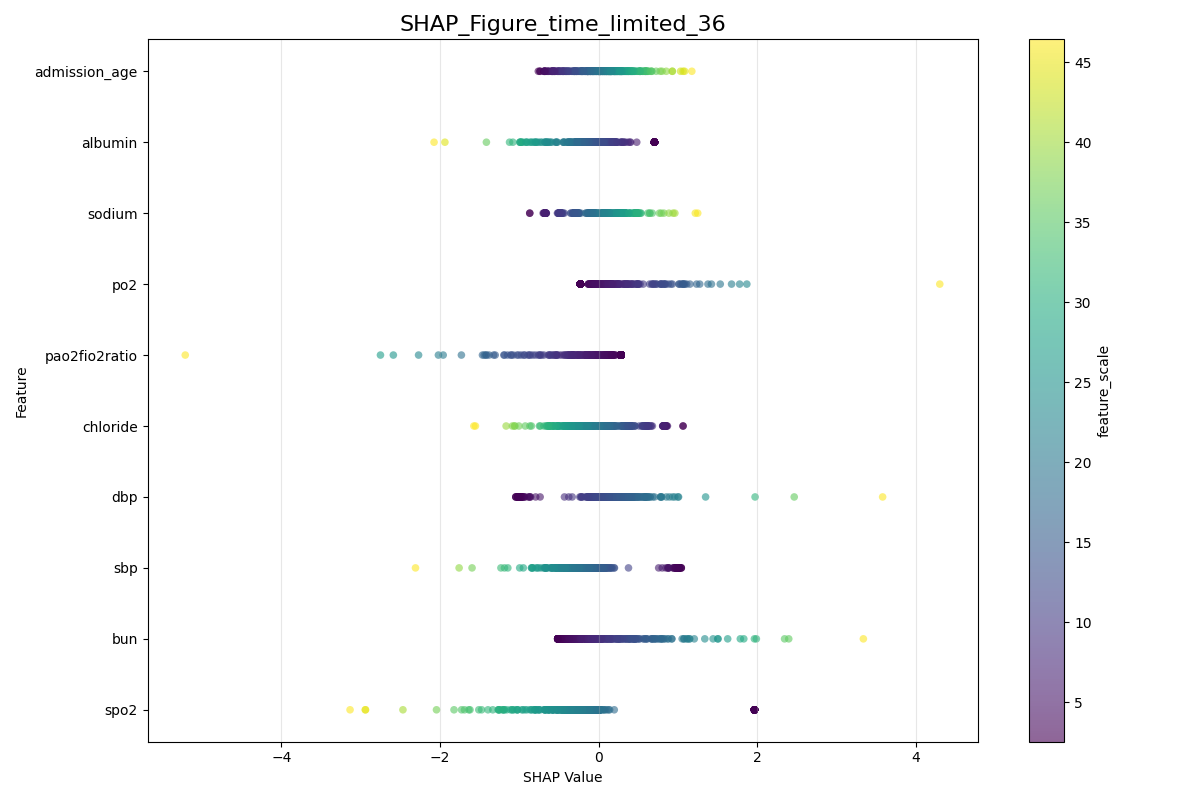


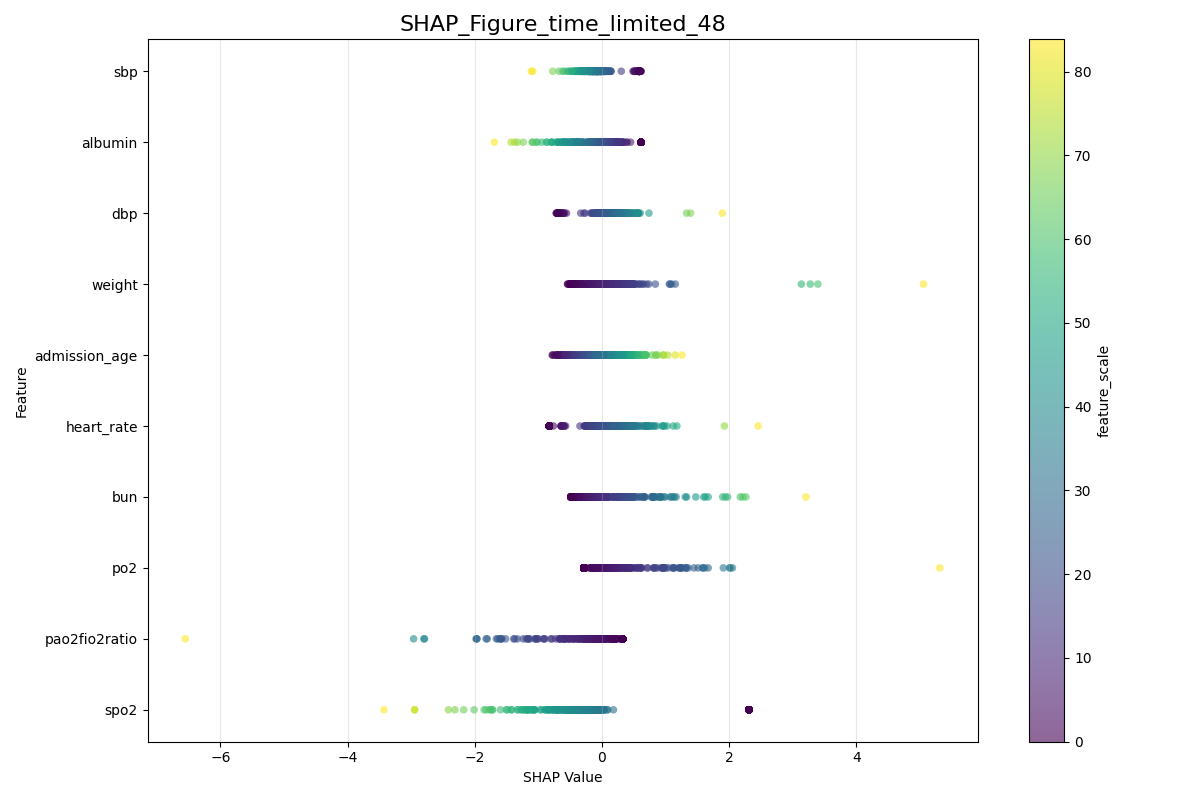


1. MIMIC-III


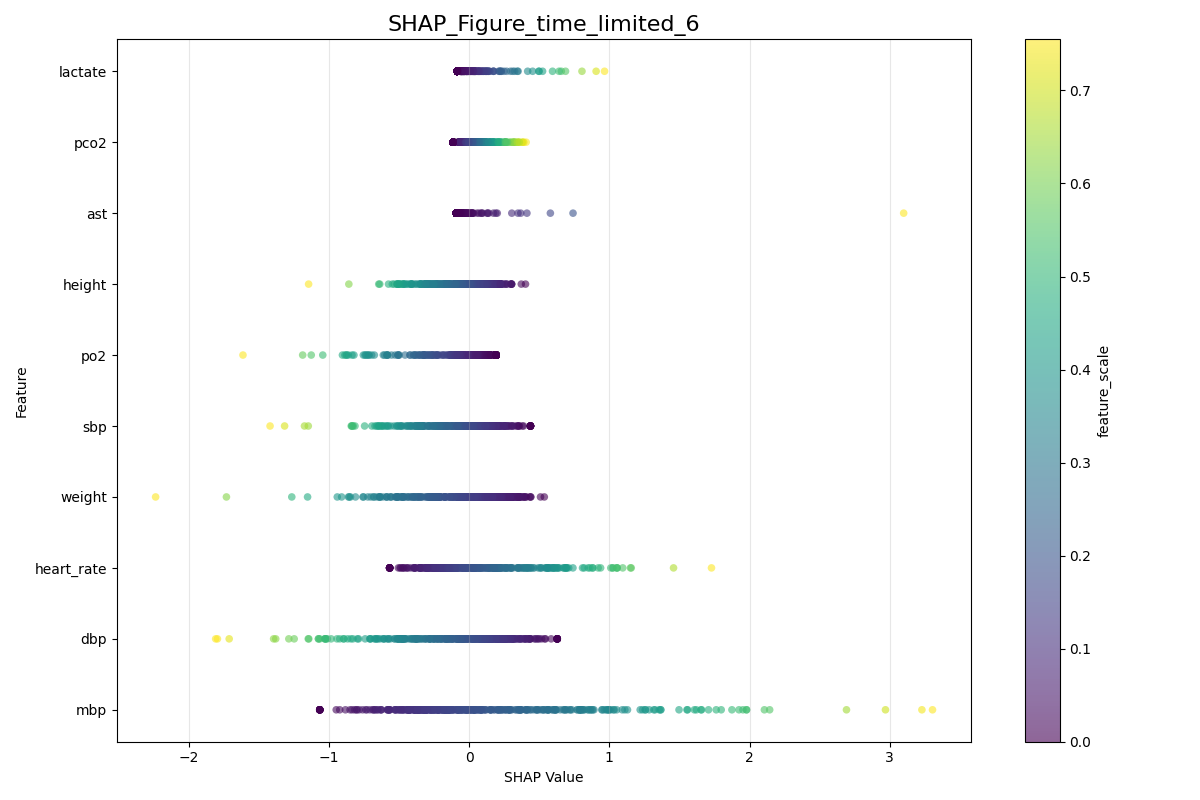


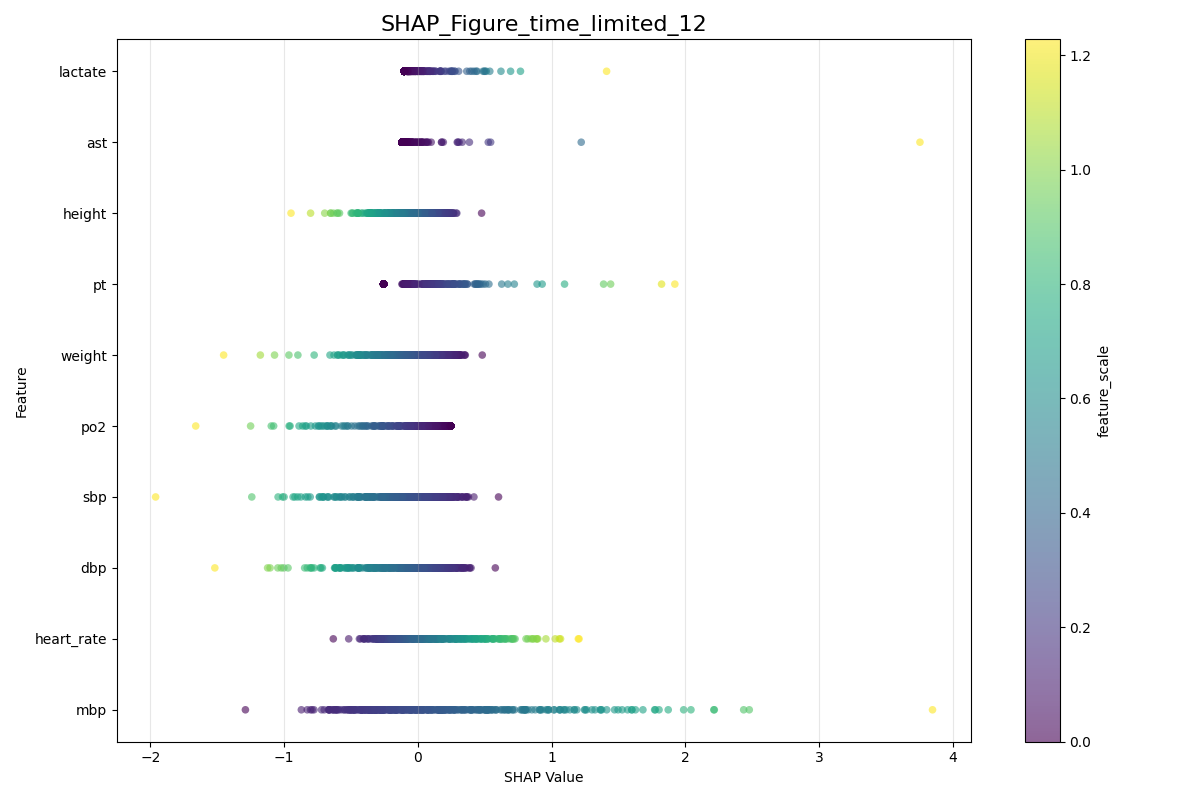


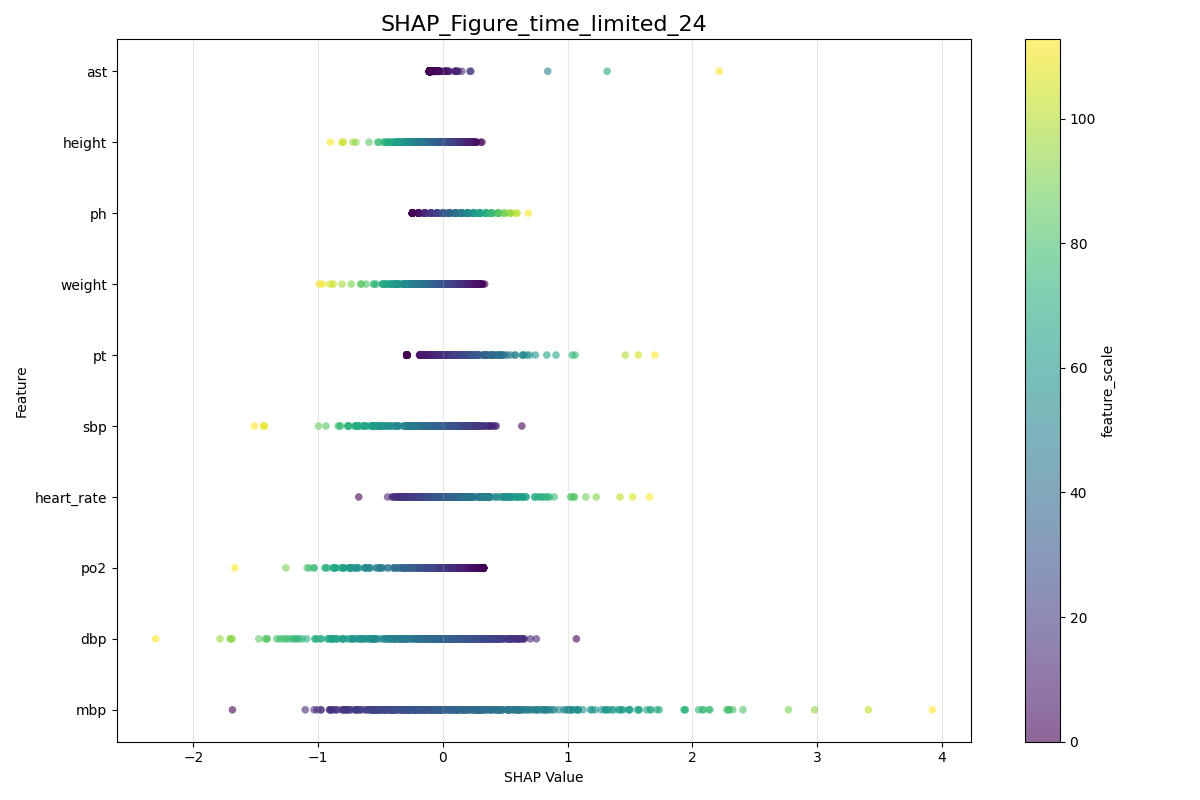


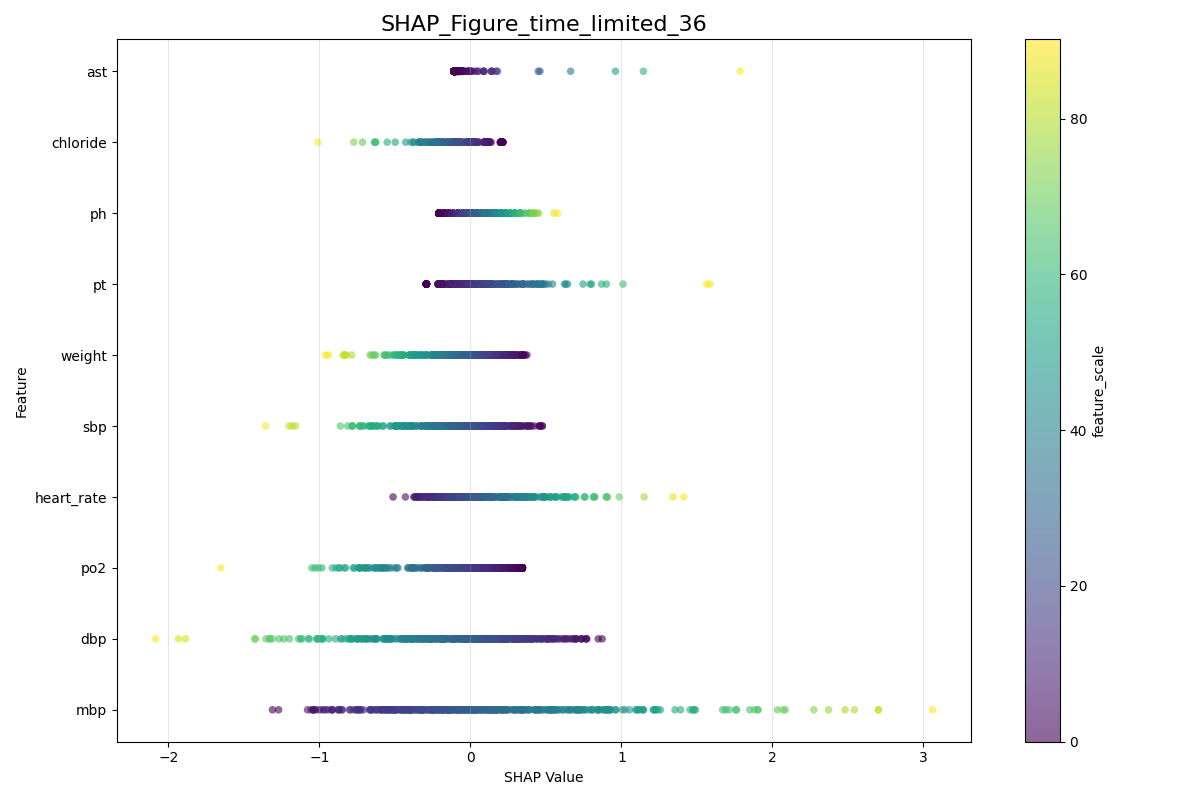


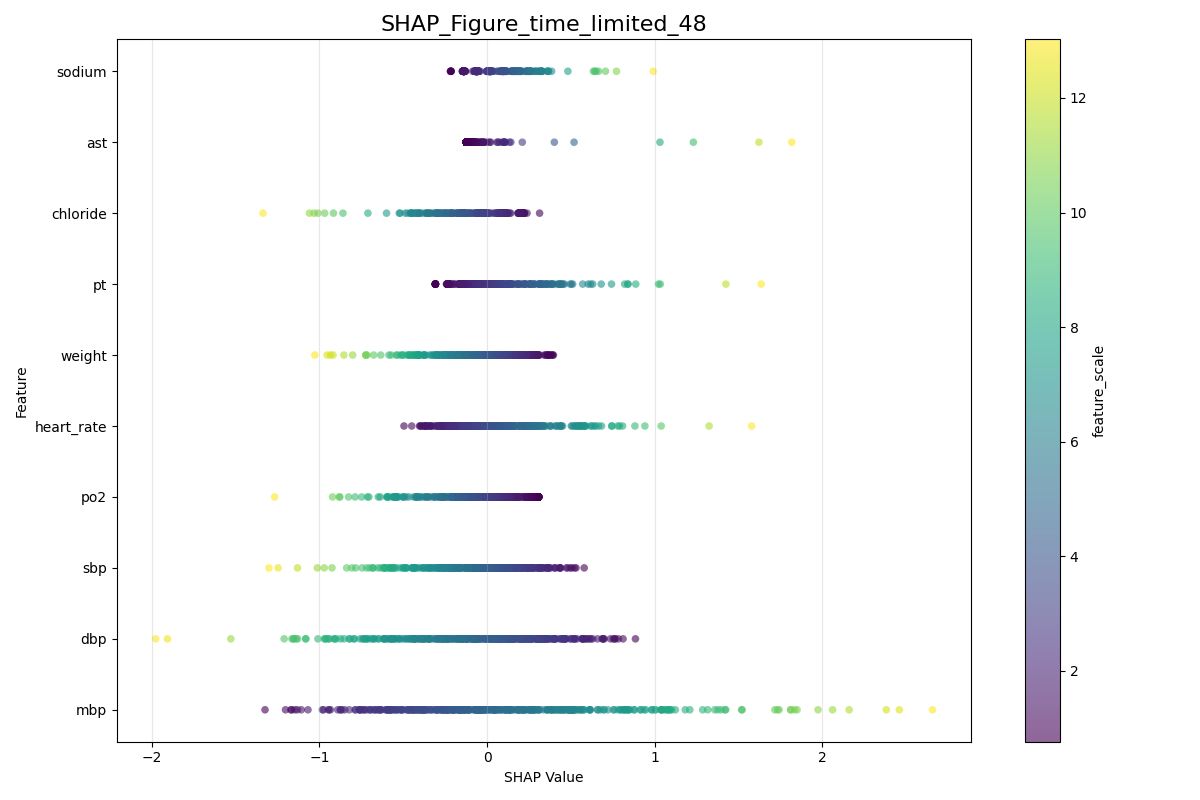


1. MIMIC-IV *ICD-9*


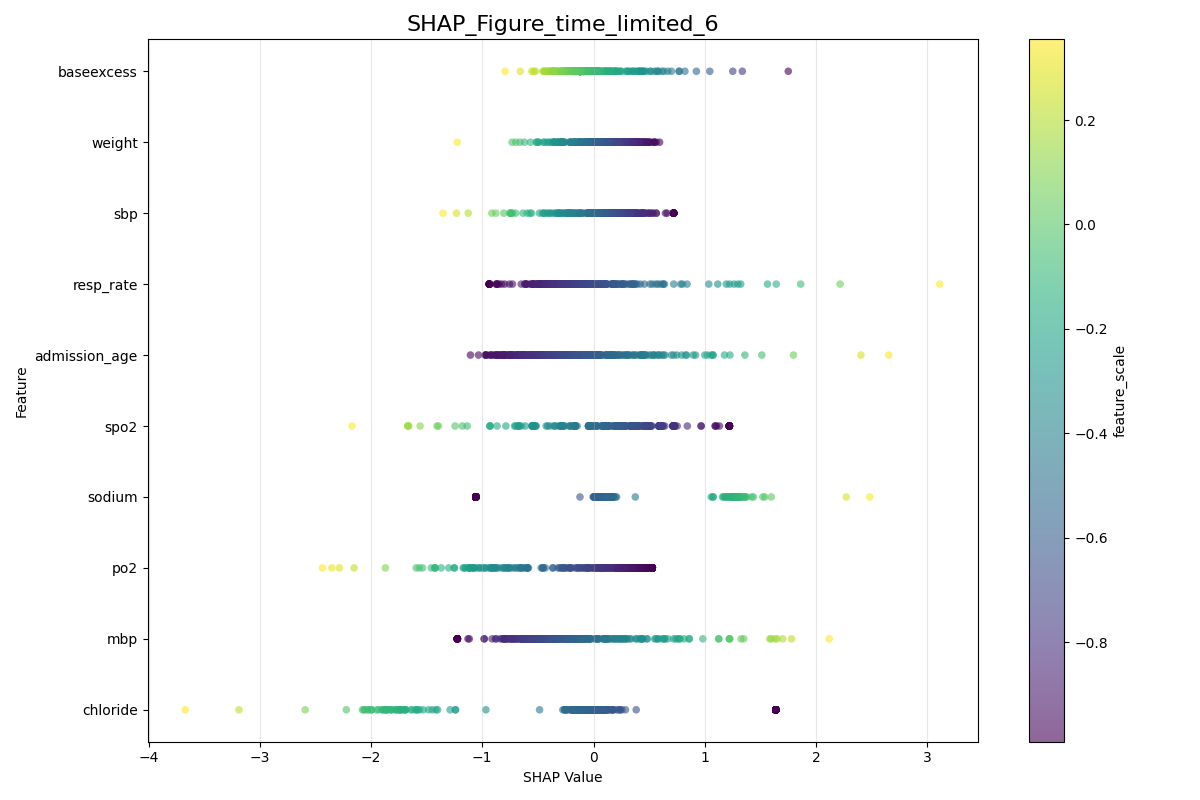


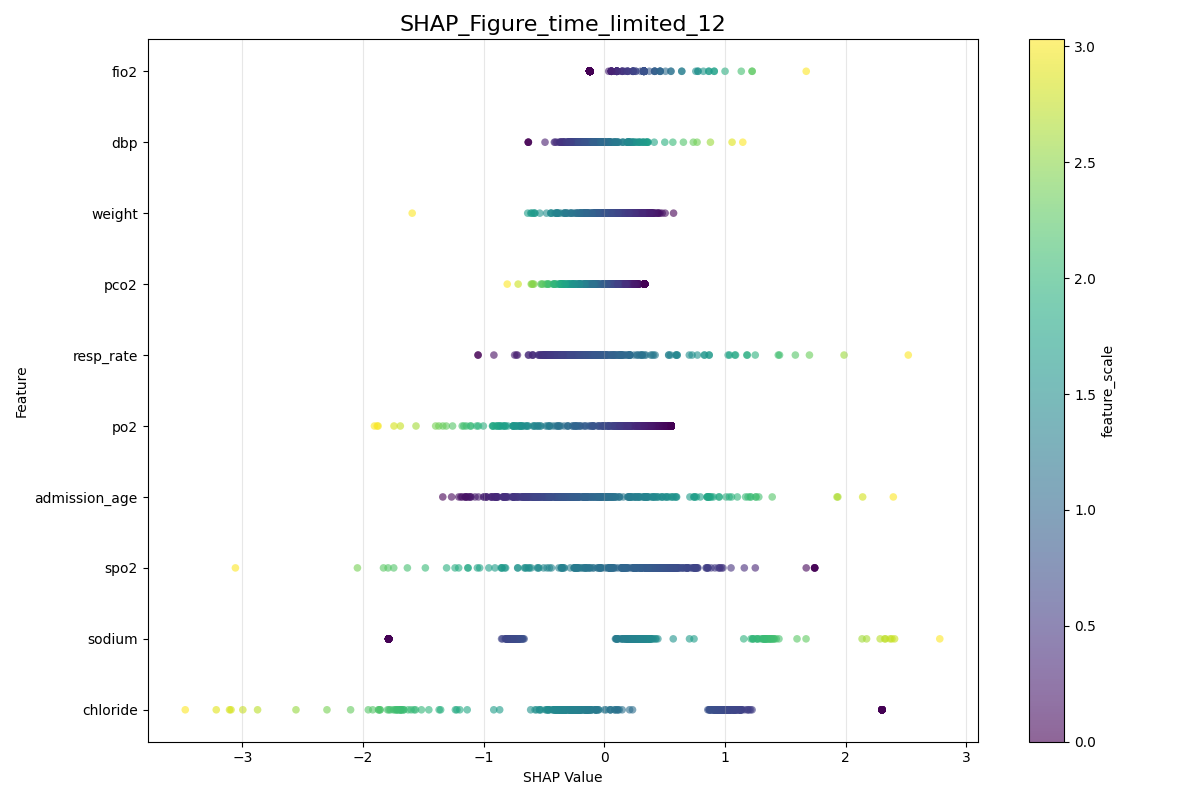


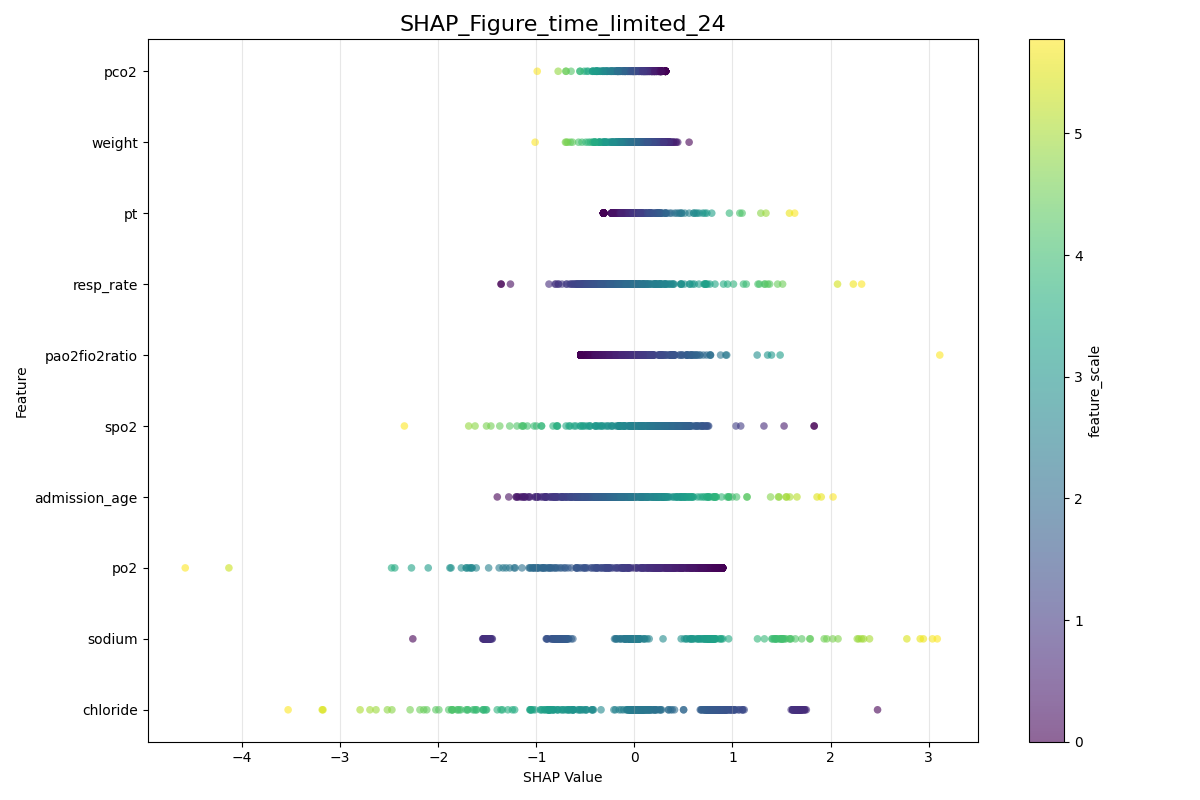


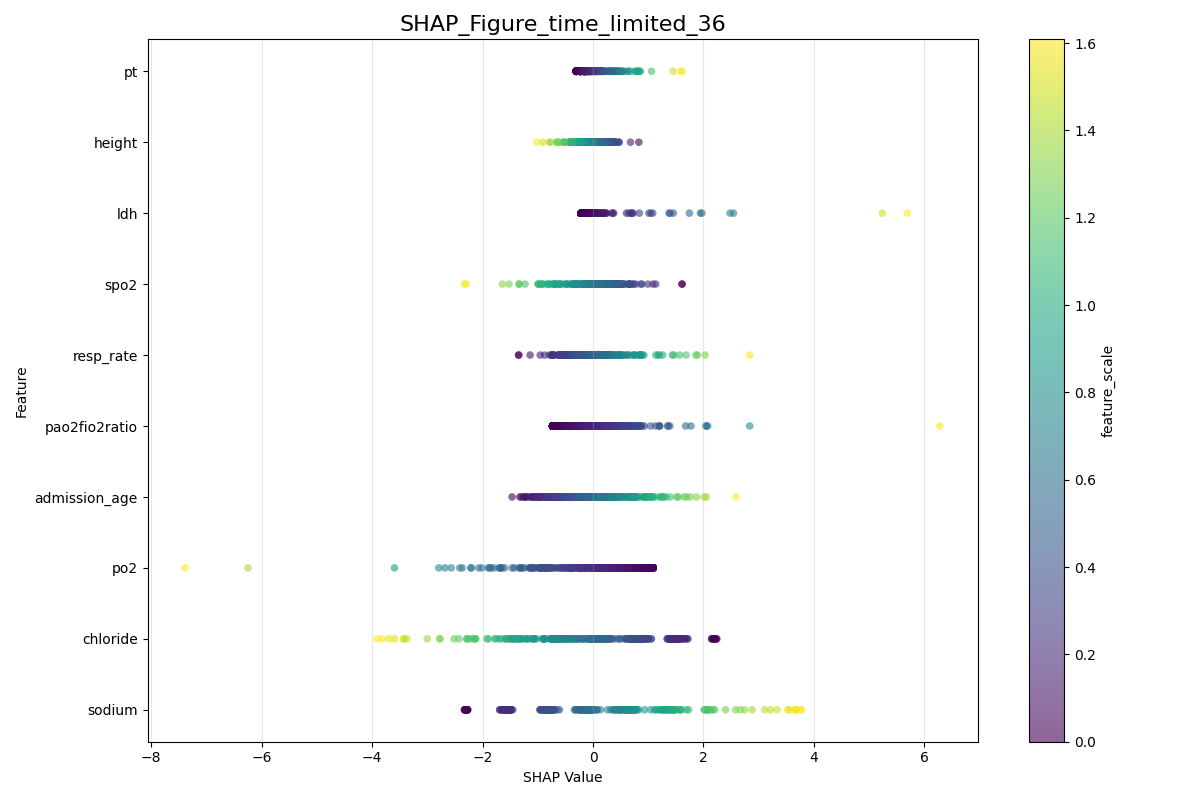


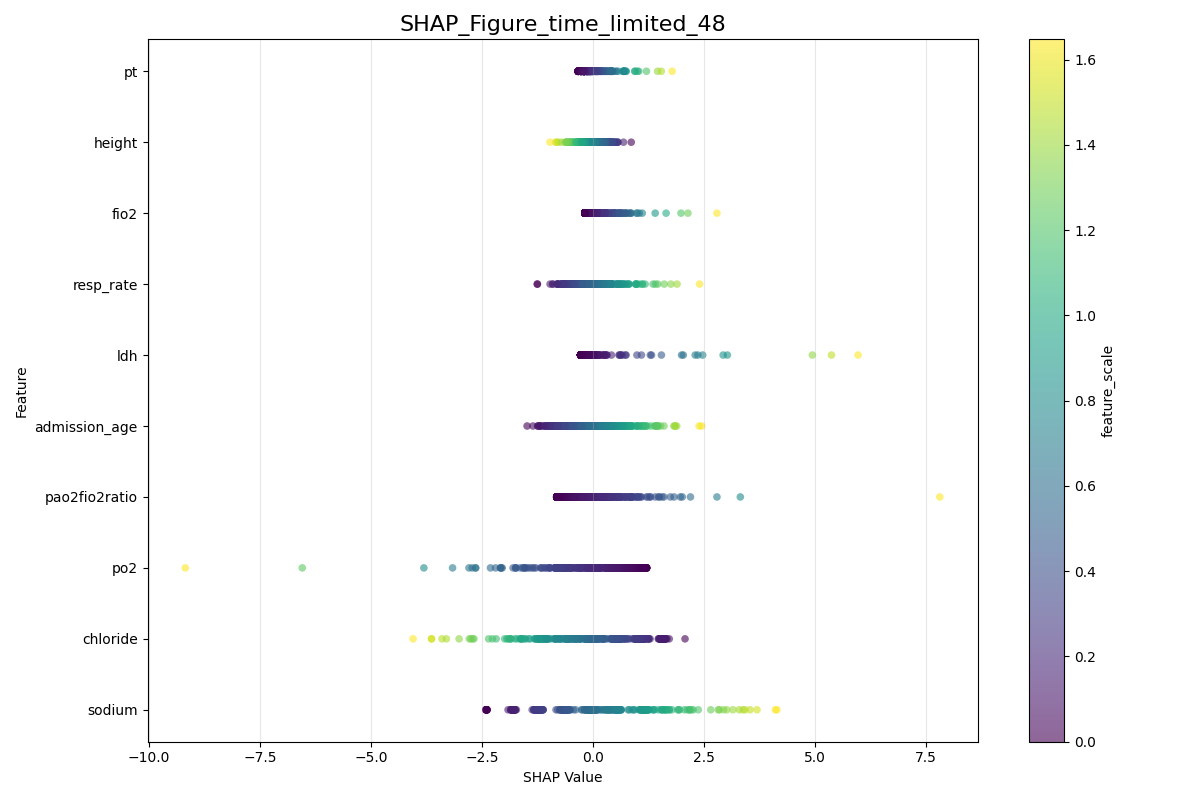


1. MIMIC-IV *ICD-10*


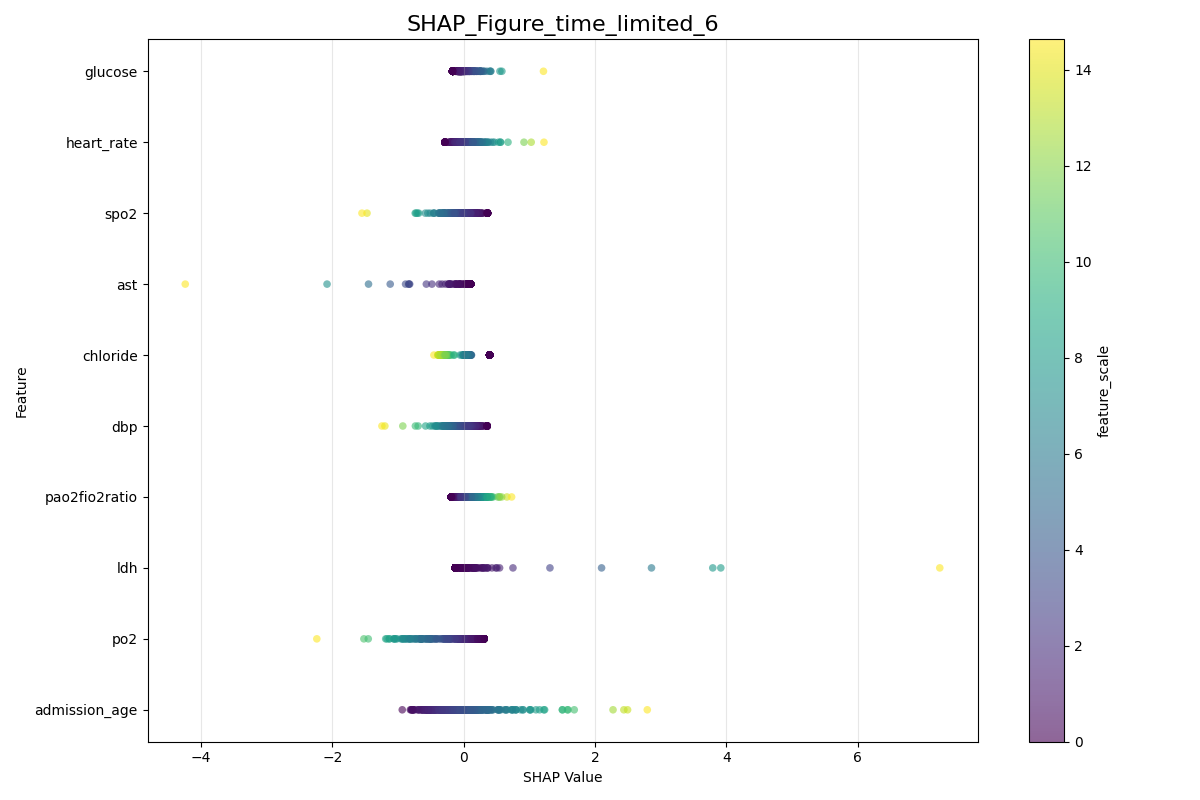


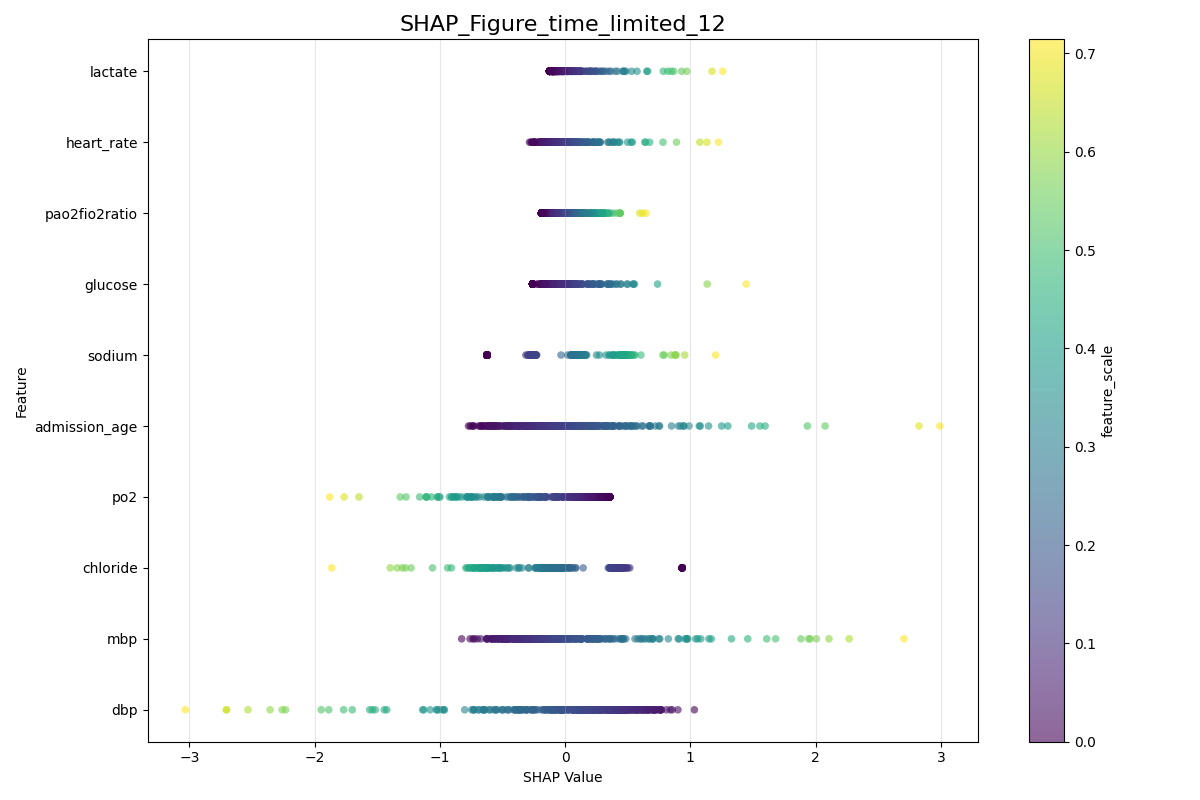


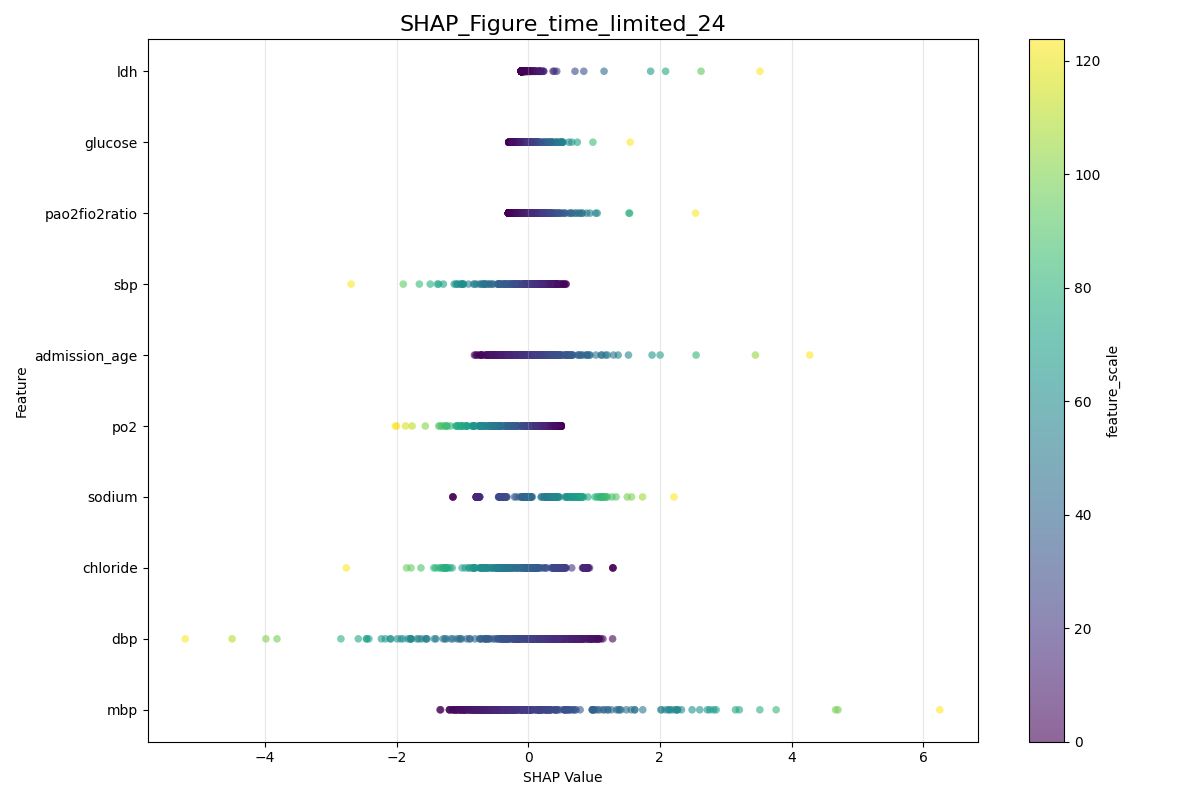


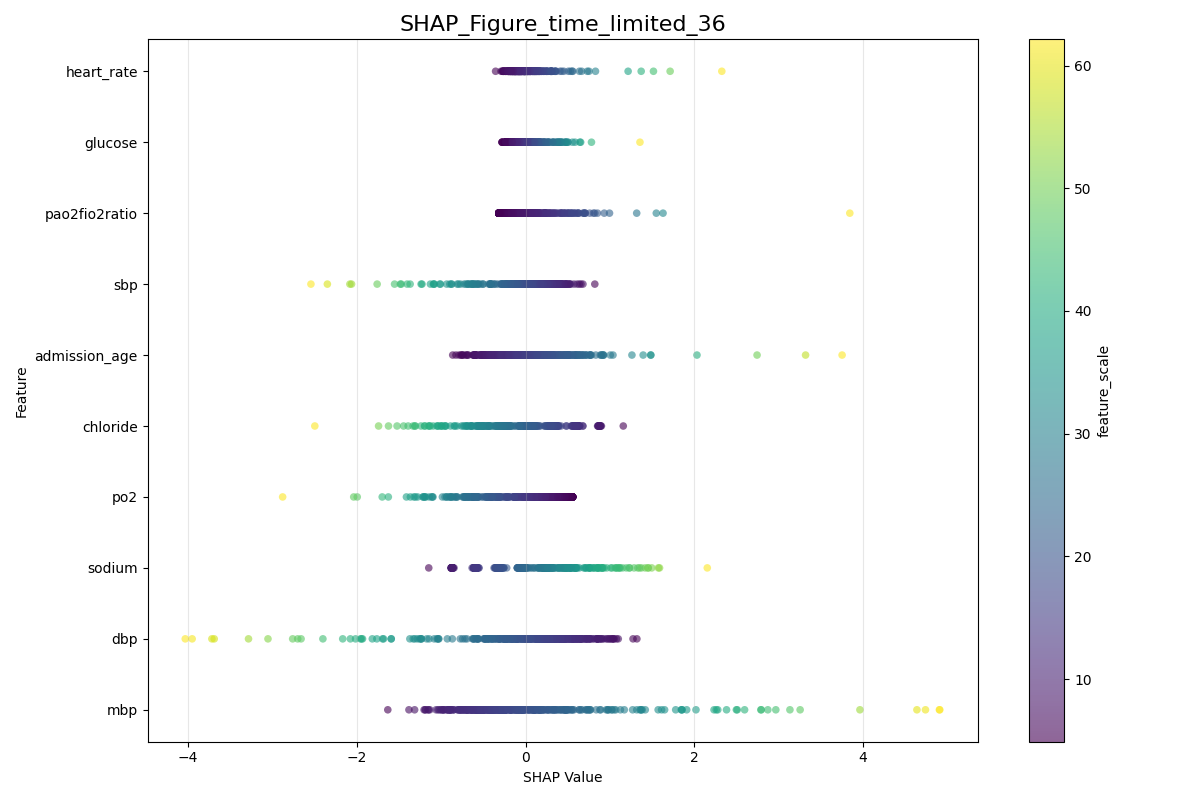


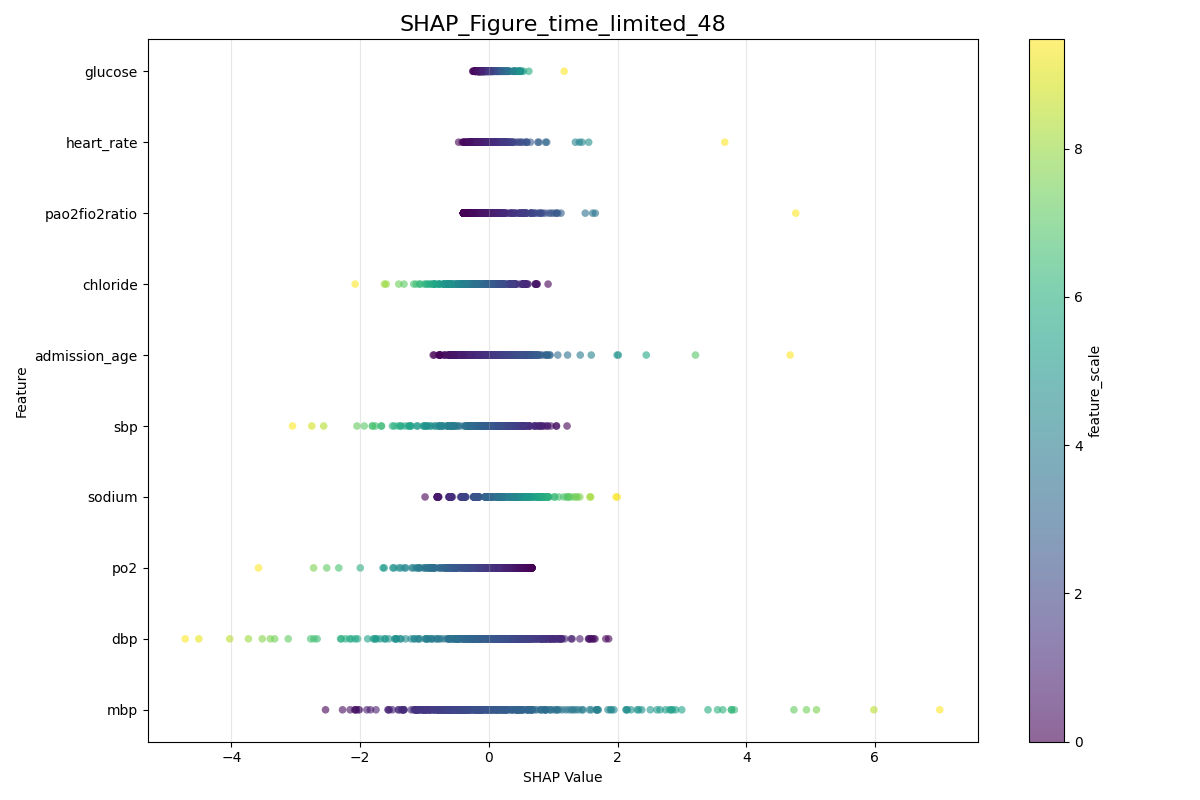

Supplement: Multimedia Appendix 4 [file medinform-v14-e81145-s004.docx]
