# Supplementary material for: Graph Network Feature Space Fusion for Predicting Irregularly Sampled Medical Time-Series Data: Deep Learning Model Development and Validation Study
Source: JMIR Med Inform. 2026 Jul 3;14:e81145. doi: 10.2196/81145 (PMC13331332; doi:10.2196/81145)
Supplement: Multimedia Appendix 5 [file medinform-v14-e81145-s005.docx]

1. Mortality Delong Test

| **Compare_method** | **Mimic4_icd9** | **Private data** |
| --- | --- | --- |
| Our work compare with FCN | 0.000563 | 0.003017 |
| Our work compare with TCN | 0.000911 | 0.003516 |
| Our work compare with Nbeats | 0.000953 | 0.004187 |
| Our work compare with Crossformer | 0.000874 | 0.003337 |
| Our work compare with Dsformer | 0.000719 | 0.004539 |
| Our work compare with T-Lstm | 0.000746 | 0.002108 |
| Our work compare with Grud | 0.000774 | 0.002974 |
| Our work compare with mTAND | 0.000815 | 0.002559 |
| Our work compare with ContiFormer | 0.000882 | 0.002517 |

1. Regression Prediction U-tests

| **Compare_method** | **Mimic4_icd9** | **Private data** |
| --- | --- | --- |
| Our work compare with FCN | 0.001784 | 0.004063 |
| Our work compare with TCN | 0.001905 | 0.003981 |
| Our work compare with Nbeats | 0.002362 | 0.003887 |
| Our work compare with Crossformer | 0.002154 | 0.003674 |
| Our work compare with Dsformer | 0.002237 | 0.004031 |
| Our work compare with T-Lstm | 0.001553 | 0.003906 |
| Our work compare with Grud | 0.001776 | 0.004105 |
| Our work compare with mTAND | 0.001615 | 0.004002 |
| Our work compare with ContiFormer | 0.001973 | 0.003791 |
